# Association of substance use with suicide mortality: An updated systematic review and meta-analysis

**DOI:** 10.1016/j.dadr.2024.100310

**Published:** 2024-12-13

**Authors:** Alison Athey, Jaimie Shaff, Geoffrey Kahn, Kathryn Brodie, Taylor C. Ryan, Holly Sawyer, Aubrey DeVinney, Paul S. Nestadt, Holly C. Wilcox

**Affiliations:** a The RAND Corporation, United States; b Johns Hopkins Bloomberg School of Public Health, United States; c Henry Ford Health, United States; d Johns Hopkins School of Medicine, United States; e University of Washington School of Public Health, United States; f Westat, United States

**Keywords:** Suicide, Substance use, Alcohol, Opioids, Cannabis, Meta-analysis

## Abstract

**Background:**

Rates of suicide mortality and substance use have increased globally. We updated and extended existing systematic reviews of the association between substance use and suicide.

**Methods:**

This systematic review and meta-analysis explored the association between substance use and suicide mortality in peer reviewed, longitudinal cohort studies published from 2003 through 2024. Risk of bias was assessed using the Newcastle-Ottawa Scale. Pooled data were analyzed using a quality effects model. Meta-regression was used to assess the effect of moderation by study quality. Asymmetry in funnel plots and Doi plots were used to detect reporting bias.

**Findings:**

The analysis involved 47 studies from 12 countries. Substance misuse (SMR: 5.58, 95 % CI: 3.63–8.57, I^2^: 99 %) was significantly associated with risk for suicide. Alcohol (SMR: 65.39, 95 % CI: 3.02–19.62, I^2^: 99 %), tobacco (SMR: 1.83, 95 % CI: 1.20–2.79, I^2^: 83 %), opioid (SMR: 5.46, 95 % CI: 3.66–8.15, I^2^: 96 %), cannabis (SMR 3.31, 95 % CI: 1.42–7.70, I^2^: 95 %), and amphetamine (SMR 11.97, 95 % CI: 3.13–45.74, I^2^: 99 %) misuse were each linked to higher rates of suicide mortality. The association between substance misuse and suicide was stronger for females (SMR: 12.37, 95 % CI: 7.07–21.63, I^2^: 98 %) than males (SMR: 5.21, 95 % CI: 3.09–8.78, I^2^: 99 %) overall and in analyses of specific substances. Further disaggregated data were not available to sufficiently explore for potential health inequities across social factors.

**Conclusions:**

This meta-analysis highlights that substance misuse remains a significant suicide risk factor. It underscores the need for universal and targeted prevention and equitable access to effective interventions.

## Highlights

• People who misuse substances are at 5.58 times higher risk for suicide than controls.
• The association between substance use and suicide was stronger in females than males.
• Alcohol, cannabis, opioids, amphetamines, and tobacco misuse increase suicide risk.
• A lack of disaggregated data limits the field from identifying potential health inequities.

## 1 Introduction

Globally, over 700,000 people die by suicide each year. Suicide is a leading cause of death, particularly for those under the age of 35 (Ilic and Ilic, 2022) and recent data show concerning increases in suicide-related disparities across social factors including racial/ethnic identity, age, gender identity, sexuality, and geography (Perry et al., 2022). A growing body of evidence suggests both a distal and proximal link between substance misuse and suicide (Borges et al., 2017, Esang and Ahmed, 2018). Meta-analyses of psychological autopsy studies show that the risk of suicide was three-fold higher for those with substance use disorder (Favril et al., 2022). Substance misuse and substance use disorders are conceptualized as a potentially modifiable risk factor for suicide (Mclellan, 2017) and health inequities related to substance use and substance-related mortality are well established (CDC, 2022, McCuistian et al., 2021).

Substance misuse includes the use of illicit substances and the use of licit substances in inappropriate situations or amounts. Substance misuse may play a precipitating role in suicide. Approximately one third of people who die by suicide have alcohol or drugs in their system at the time of death (Choi et al., 2018). Acute substance misuse or intoxication can result in a state of disinhibition, impulsivity, and impaired judgment that can precipitate suicidal behaviors in vulnerable individuals (Turecki and Brent, 2016). Moreover, both persistent and binge use of substances can be linked to family disruption, occupational and financial stressors, and legal challenges as well as personality traits and mental disorders that may increase vulnerability to suicide (Yuodelis-Flores and Ries, 2015)^.^ Acute substance misuse may be considered a risk factor for suicide.

Substance use that becomes risky, uncontrollable, results in social impairment, requires higher and higher doses, and/or leads to symptoms of withdrawal may be diagnosed as Substance Use Disorder (SUD; American Psychiatric Association, 2022). Chronic substance use may be related to cognitive and executive dysfunction and is seen as a developmental or mediating factor associated with suicide risk (Fernández-Serrano et al., 2010, Maharjan et al., 2022, Turecki and Brent, 2016). Additionally, people who discontinue medication assisted treatment for opioid use disorder are at elevated risk for suicide (Padmanathan et al., 2022), in part due to symptoms of dysphoria that accompany withdrawal. Substance use disorders may also exacerbate the impact of acute substance misuse on suicide risk. Some evidence suggest that people with alcohol use disorders and low levels of depression have similar suicide attempt histories to people with severe depression (Mitchell et al., 2023). The proximal risk for suicide associated with nonalcohol substance use disorder is seven-fold higher than in those without substance use disorders (Conner et al., 2019). Substance use disorders may represent a suicide warning sign and may also precipitate risk for suicide.

Substance misuse is a growing challenge globally. The 2022 World Drug Report estimated a 26 % increase in global drug use over the past decade amidst an environment of evolving drug policies, increasing drug production and trafficking, and inequitable access to care and treatment (United Nations Office on Drugs and Crime, 2022). The COVID-19 pandemic was also associated with increases in substance misuse, co-occurring mental health challenges, and widening health inequities (Panchal et al., 2023, Roberts et al., 2021). Further, the types of commonly misused substance have changed in the last decades. Rates of cannabis use have increased in regions where policies regulating the substance has changed (United Nations Office on Drugs and Crime, 2022) and changes in the composition of illicit opioids have driven shifts in the opioid overdose crisis (Ciccarone, 2019). In light of this evolving context, updated estimates of the relationship between substance use and suicide, inclusive of estimates by available social determinants of health, are needed.

This study updates and augments the classic 1997 empirical review by Harris and Barraclough, as well as the 2004 update by Wilcox and colleagues. Harris and Barraclough’s (1997) review focused on a broad range of psychiatric disorders and used a MEDLINE search to identify papers until 1993 and a read through of The Lancet, British Medical Journal, New England Journal of Medicine, British Journal of Psychiatry, Psychological Medicine, Archives of General Psychiatry, and Acta Psychiatrica Scandinavica to include articles through mid-1995. Wilcox and colleague’s (2004) review served to update Harris and Barraclough’s study with a focus on alcohol and drug disorders and included studies through 2002 as identified through an enhanced MEDLINE search utilizing medical subject heading (MeSH) search strings for all papers until 2002, and a read through of the journals read by Harris and Barraclough as well as Addiction, Alcoholism: Clinical and Experimental Research, Drug and Alcohol Dependence, and Journal of Studies on Alcohol. Both studies also included papers identified by reviewing the reference sections of papers identified for inclusion (Harris and Barraclough, 1997, Wilcox et al., 2004). The updated review by Wilcox and colleagues (2004) also identified an additional category of drug use, intravenous drug use, given the influx of HIV-related research at the time of the study. In totality, the findings summarized by Wilcox (2004) found the following estimated standardized mortality rates for suicide: alcohol use disorder: 9.79 (95 % CI: 8.98, 10.65); opioid use disorder: 13.51 (95 % CI: 10.47, 17.15); intravenous drug use: 13.73 (95 % CI: 10.29, 17.96); mixed drug use: 16.85 (95 % CI: 14.73, 19.20); heavy drinking: 3.51 (95 % CI: 2.51, 4.78). Wilcox (2004) found greater Standardized Mortality Ratios (SMR) among females for alcohol use disorder, and, while sex-stratified SMRs were reported, there were few observed results for the other categories, highlighting the limited amount of demographic information provided in included studies to assess for potential inequities by sex.

An updated meta-analysis evaluating the association between substance misuse and suicide is indicated because of shifting substance use patterns, treatment, and policy. This study builds from these previous studies by updating both the methods and approach in multiple ways. First, this updated review expands the search to databases in addition to MEDLINE, as multiple databases have been established in the previous two decades and advances in technology allow more rapid indexing of literature. Second, this study follows the approach of Wilcox and colleagues (2004) to capture additional substances and use types included in literature since the prior studies. Third, this study uses an enhanced methodology to differentiate the association between suicide and 1) different substances, 2) different types of substance use (e.g., any substance use, substance misuse, and substance use disorders), and 3) licit and illicit substances, reflecting current understanding of the complexity of substance use. Fourth, to assess potential health inequities, this study expanded upon the sex-stratification used by Wilcox and colleagues (2004) to include key demographics such as race, ethnicity, gender identity, sexuality, and geography. Thus, this study aimed to provide updated and more comprehensive estimates of the association between substance use and suicide mortality across social factors and in the context of increased and changing patterns of substance misuse.

## 2 Materials and methods

### 2.1 Search strategy and selection criteria

This systematic review and meta-analysis replicated the strategy defined by Wilcox and colleagues (2004) to maintain consistency. With the help of a research librarian, we selected search terms that conformed to the indexing systems of each database (see Supplemental Table 1.) Broadly, we used medical subject heading (MeSH) and similar terms for (1) suicide, (2) substance-related disorders and specific substances, (3) death, (4) and cohort studies. The search protocol was registered on PROSPERO (CRD42021264807).

Searches were conducted in PubMed, EMBASE, CINAHL, PsychINFO, and Cochrane databases. We filtered search results to include all peer-reviewed papers, written in English, and published between January 1, 2003 (i.e., the end of the search conducted by Wilcox and colleagues) and June 12, 2024. Studies were downloaded into EndNote, a citation management program, and uploaded to Covidence, a systematic review support software.

Studies were included if they: (1) were longitudinal cohort studies, (2) followed participants for > 2 years, (3) specified the observed number of suicides, and (4) provided expected values for suicide or provided sufficient data such that expected values could be estimated from the suicide rate in the non-substance using sample. Studies were excluded if: (1) > 10 % of participants were lost to follow-up and (2) did not provide necessary information to support extraction of the observed and expected number of suicide deaths.

Two independent reviewers screened the titles and abstracts, completed full-text reviews, and extracted data from included studies. Disagreements were resolved by a third independent review and discussion among reviewers. We developed, tested, and revised a data extraction form (Supplement Table 2) based on the data collected by Wilcox and colleagues (2004). In line with Wilcox and colleagues (2004), we extracted study characteristics, substance use exposures and characteristics, and the standardized mortality ratios for suicide. Specifically, we extracted data describing the composition of the population by gender (male, female, transgender, and other), racial/ethnic (White, Black, Asian/Pacific Islander, Latinx, Native/Indigenous, Other, and Multi-racial), sexual orientation (heterosexual, bisexual, homosexual, other, unknown), and geography (i.e., country or countries from which the cohort was drawn.) We also extracted disaggregated substance use characteristics and suicide outcomes for these minoritized groups. Where SMRs were not given in the study they were calculated based on the available information. Mortality rates or the absolute number and sample size in the control population were used to calculate a suicide rate that was then multiplied by the case population to estimate the expected number of deaths. This number along with the observed number of case deaths was used to calculate the SMR. Definitions for “use” and “misuse” varied between studies and substances; we extracted data based on the definitions provided in individual studies.

We used the Newcastle-Ottawa Scale (NOS; Wells et al., 200); Supplement Table 3) for assessing the quality of nonrandomized studies in meta-analyses. The NOS scores studies on eight different domains to assess for potential biases across the research process (Wells et al., 2009). Domains include: representativeness of the exposed cohort, selection of the non-exposed cohort, ascertainment of exposure, demonstration that the outcome of interest was not present at the start of the study, comparability of the cohorts on the basis of the design or analysis, assessment of the outcome, whether follow up time was long enough for outcomes to occur, and whether the size of the follow-up cohorts were large enough (Wells et al., 2009). Data abstractors independently assessed each study per the NOS, and discrepancies were resolved by a third rater.

### 2.2 Data analysis

Pooled SMR estimates were estimated using the quality effects (QE) model proposed by Doi and colleagues (Doi et al., 2015, Furuya-Kanamori et al., 2018). Data analyses were conducted using MetaXL version 5.3 (MetaXL (epigear.com).

We used meta-regression to assess the effect of moderation by study quality (i.e., NOS score). As recommended by Doi and colleagues (2015), the transformed effect sizes were regressed on NOS score using a linear model weighted by the QE weights. MetaXL was used to create and export the transformed effect sizes and weights, and the regression was run in R version 4.2.

Asymmetry in funnel plots and Doi plots (Doi et al., 2015, Furuya-Kanamori et al., 2018) was assessed, and the presence of asymmetry was taken as evidence of reporting bias as, in the absence of bias, one would expect individual study estimates to be equally likely to be above and below the pooled estimate. We followed the GRADE (Grading of Recommendations, Assessment, Development, and Evaluations) process for summarizing the overall strength of the evidence (Guyatt et al., 2008).

### 2.3 Role of the funding source

This work was supported by an NIH Training Award (T32 MH 014592, PI: Volk) and an NIH Career Development Award (K23 DA055693, PI: Nestadt). The funder of the study had no role in study design, data collection, data analysis, data interpretation, or writing of the report.

## 3 Results

Our search yielded 10,866 unique studies. A PRISMA diagram (Page et al., 2021) can be found in the supplement (Supplemental Figure 1). Following title and abstract review and subsequent full-text review, 10,819 studies were excluded per inclusion and exclusion criteria. Ultimately, we included 47 studies (Akechi et al., 2006, Arendt et al., 2013, Björkenstam et al., 2020, Bjornaas et al., 2008, Bøe et al., 2023, Bohnert et al., 2017, Bohnert et al., 2014, Bowden et al., 2018, Chang et al., 2022, Chang et al., 2017, Chung et al., 2022, Clapperton et al., 2024, Crump et al., 2021, Edwards et al., 2020, Flensborg-Madsen et al., 2009, Girardi et al., 2022, Haver et al., 2009, Hemmingsson and Kriebel, 2003, Hesse et al., 2020, Holmstrand et al., 2015, Høye et al., 2021, Hung et al., 2015, Kauppila et al., 2022, Kim et al., 2017, Lähteenvuo et al., 2021, LeardMann et al., 2013, Lee et al., 2021, Lundgren et al., 2022, Mattisson et al., 2011, Nordentoft et al., 2011, Padmanathan et al., 2022, Pan et al., 2014, Park et al., 2019, Pavarin, 2008, Pavarin et al., 2020, Pavarin et al., 2019, Pavarin et al., 2017, Pavarin and Fioritti, 2018, Pierce et al., 2015, Price et al., 2009; Riala, 2007; Ryb et al., 2006; Schneider et al., 2011; Suominen et al., 2004; Tidemalm et al., 2008; Veldhuizen and Callaghan, 2014; Yi et al., 2016) that described the association between suicide mortality and substance use in 12 countries, including 19 studies that stratified by sex (Table 1). No studies disaggregated results by gender identity or sexual orientation, and few reported results disaggregated by racial/ethnic group. SMRs and confidence intervals from the included studies and pooled estimates are presented in Table 1, Table 2, Table 3, Table 4, Table 5, Table 6, Table 7, Table 8. Results are visualized in Supplemental Figures 2–4.

**Table 1:** Study characteristics.

**Study**	**Country**	**Substance**	**Use Type**	**Sex-stratification**	**NOS Score**
Akechi et al. (2006)	Japan	Alcohol	Use	M only	5
Arendt et al. (2013)	Denmark	Cannabis	Treatment	No stratification	7
Björkenstam et al. (2020)	Sweden	Any substance	Use disorder	No stratification	9
Bjornaas et al. (2008)	Norway	Opioid	Use	M/F stratified	6
Bøe et al. (2023)	Norway	Alcohol, Any substance	Use disorder	M/F stratified	8
Bohnert et al. (2014)	USA	Tobacco	Use disorder	No stratification	7
Bohnert et al. (2017)	USA	Alcohol, Amphetamine, Any substance, Cannabis, Cocaine, Opioid, Sedative	Use disorder	M/F stratified	7
Bowden et al. (2018)	Wales	Alcohol	Misuse	No stratification	8
Chang et al. (2017)	USA, Taiwan	Opioids	Treatment	No stratification	6
Chang et al. (2022)	Taiwan	Any substance	Use disorder	No stratification	7
Chung et al. (2022)	Taiwan	Alcohol	Use disorder	M/F stratified	8
Clapperton et al. (2024)	Australia	Any substance	Use disorder	M/F stratified	5
Crump et al. (2021)	Sweden	Alcohol, Amphetamine, Any substance, Cannabis, Cocaine, Opioids	Use disorder	M/F stratified	8
Edwards et al. (2020)	Sweden	Alcohol	Use disorder	M/F stratified	7
Flensborg-Madsen et al. (2009)	Denmark	Tobacco	Use	No stratification	7
Girardi et al. (2022)	Italy	Any substance	Use disorder	No stratification	8
Haver et al. (2009)	Sweden	Alcohol	Use disorder	F only	5
Hemmingsson and Kriebel, (2003)	Sweden	Tobacco	Use	M only	6
Hesse et al. (2020)	Denmark	Any substance	Use disorder	M/F stratified	6
Holmstrand et al. (2015)	Sweden	Alcohol	Use disorder	M/F stratified	8
Høye et al. (2021)	Norway	Any substance	Use disorder	No stratification	4
Hung et al. (2015)	Taiwan	Alcohol	Use disorder	M/F stratified	6
Kauppila et al. (2022)	Mixed Icelandic	Any substance	Use disorder	No stratification	6
Kim et al. (2017)	South Korea	Any substance	Use disorder	M/F stratified	7
Lähteenvuo et al. (2021)	Finland	Any substance	Use disorder	No stratification	6
LeardMann et al. (2013)	USA	Alcohol	Misuse	No stratification	4
Lee et al. (2021)	Taiwan	Methamphetamine	Use disorder	M/F stratified	7
Lundgren et al. (2022)	Sweden	Opioids	Use	No stratification	8
Mattisson et al. (2011)	Sweden	Alcohol	Use disorder	No stratification	8
Nordentoft et al. (2011)	Denmark	Any substance	Use disorder	M/F stratified	7
Padmanathan et al. (2022)	.England	Opioids	Treatment	M/F stratified	6
Pan et al. (2014)	Taiwan	Opioid	Use disorder	M/F stratified	5
Park et al. (2019)	South Korea	Opioid	Use disorder	No stratification	8
Pavarin, (2008)	Italy	Cocaine	Use disorder	No stratification	4
Pavarin et al. (2017)	Italy	Opioid	Treatment	M/F stratified	5
Pavarin and Fioritti, (2018)	Italy	Cocaine	Use disorder	No stratification	5
Pavarin et al. (2019)	Italy	Opioid	Treatment	M/F stratified	6
Pavarin et al. (2020)	Italy	Alcohol, Cocaine, Any substance	Treatment	M/F stratified	6
Pierce et al. (2015)	England	Opioid	Use	No stratification	5
Price et al. (2009)	Sweden	Cannabis	Use	No stratification	5
Riala, 2007	Finland	Tobacco	Use	M/F stratified	6
Ryb et al. (2006)	USA	Alcohol	Use	No stratification	6
Schneider et al. (2011)	Germany	Alcohol, Tobacco	Use, Misuse	No stratification	6
Suominen et al. (2004)	Finland	Any substance	Use disorder	No stratification	6
Tidemalm et al. (2008)	Sweden	Alcohol, Any substance	Use disorder	M/F stratified	7
Veldhuizen and Callaghan, (2014)	USA	Opioid	Use disorder	M/F stratified	6
Yi et al. (2016)	South Korea	Alcohol	Use, Misuse	No stratification	6

**Table 2:** Association between alcohol use and suicide.

**Study**	**Country**	**Time**	**N suicides**		**SMR (95 % CI)**
			**Observed**	**Expected**	
**Overall**					
Bøe et al. (2023)**	Norway	2008–2018	69	84.1	0.82 (0.64–1.03)
Bohnert et al. (2017)	USA	2006–2011	1259	516.82	2.44 (2.30–2.57)
Bowden et al. (2018)	Wales	2006–2011	125	15.88	7.87 (6.55–9.31)
Chung et al. (2022)	Taiwan	2001–2016	3478	464.8	7.48 (7.24–7.73)
Crump et al. (2021)	Sweden	2003–2016	1034	N/A	5.51 (5.15–5.89)
Edwards et al. (2020)	Sweden	1950–2012	4387	717	6.12 (5.94–6.30)
Holmstrand et al. (2015)	Sweden	1947–2011	29	1.28	22.66 (15.16–31.65)
Hung et al. (2015)	Taiwan	1985–2008	65	N/A	21.2 (16.0–26.3)
LeardMann et al. (2013)	USA	2001–2008	25	9.19	2.72 (1.76–3.89)
Mattisson et al. (2011)	Sweden	1947–1997	27	3.95	6.84 (4.50–9.66)
Pavarin et al. (2020)	Italy	1975–2016	37	N/A	5.17 (3.74–7.13)
Ryb et al. (2006)*	*USA*	*1983–1995*	*34*	*35*	*0.97 (0.67–1.33)*
Schneider et al. (2011)a*	*Germany*	*1984–2003*	*28*	*27.86*	*1.01 (0.67–1.41)*
Schneider et al. (2011)*b*	Germany	1984–2003	10	4.82	2.07 (0.99–3.56)
Tidemalm et al. (2008)	Sweden	1973–2003	357	282.24	1.26 (1.14–1.40)
Yi et al. (2016)a*	*South Korea*	*1985–2008*	*33*	*12.5*	*2.64 (1.82–3.62)*
Yi et al. (2016)b	South Korea	1985–2008	24	7.16	3.35 (2.15–4.83)
Total					5.39 (3.02–9.62) I^2^ = 99 %
Total (misuse)					5.51 (3.12–9.74) I^2^ = 99 %
Total (any use)*					*1.39 (0.73–2.67)* I^2^ = 90 %
**Females only (misuse)**					
Bøe et al. (2023)**	Norway	2008–2018	22	28.2	0.78 (0.49–1.14)
Bohnert et al. (2017)	USA	2006–2011	28	5.65	4.96 (3.29–6.96)
Chung et al. (2022)	Taiwan	2001–2016	764	51.1	14.95 (13.91–16.03)
Crump et al. (2021)	Sweden	2003–2016	247	N/A	11.57 (10.13–13.22)
Edwards et al. (2020)	Sweden	1950–2012	1158	94.8	12.22 (11.52–12.93)
Haver et al. (2009)	Sweden	1981–2007	7	1.86	3.76 (1.49–7.07)
Holmstrand et al. (2015)	Sweden	1947–2011	1	0.07	14.29 (0.01–56.01)
Hung et al. (2015)	Taiwan	1985–2008	3	N/A	16.10 (0.00–34.40)
Pavarin et al. (2020)	Italy	1975–2016	N/A	N/A	8.43 (3.79–18.77)
Tidemalm et al. (2008)	Sweden	1973–2003	63	35.68	1.77 (1.36–2.23)
Total					11.54 (5.99–22.25) I^2^ = 98 %
**Males only (misuse)**					
Akechi et al. (2006)	Japan	1990–2000	116	240	0.48 (0.40–0.58)
Bøe et al. (2023)**	Norway	2008–2018	47	54.0	0.87 (0.64–1.14)
Bohnert et al. (2017)	USA	2006–2011	1231	531.62	2.32 (2.19–2.45)
Chung et al. (2022)	Taiwan	2001–2016	2714	413.7	6.56 (6.32–6.81)
Crump et al. (2021)	Sweden	2003–2016	787	N/A	4.81 (4.45–5.19)
Edwards et al. (2020)	Sweden	1950–2012	3229	622.2	5.19 (5.01–5.37)
Holmstrand et al. (2015)	Sweden	1947–2011	28	1.53	18.30 (12.15–25.71)
Hung et al. (2015)	Taiwan	1985–2008	62	N/A	21.50 (16.1–26.80)
Pavarin et al. (2020)	Italy	1975–2016	N/A	N/A	4.81 (3.38–6.84)
Tidemalm et al. (2008)	Sweden	1973–2003	294	245.65	1.19 (1.06–1.33)
Total					5.14 (2.59–10.19) I^2^ = 100 %

** Sample included only people with a prior suicide attempt

⁎ Studies which measured any or unspecified use of alcohol (as opposed to misuse, use disorder)

**Table 3:** Association between tobacco use and suicide.

**Study**	**Country**	**Time**	**N suicides**		**SMR (95 % CI)**
			**Observed**	**Expected**	
**Overall**					
Bohnert et al. (2014)	USA	2005–2008	1237	657.36	1.88 (1.78–1.99)
Flensborg-Madsen et al. (2009)*	*Denmark*	*1976–2002*	*84*	*71.2*	*1.18 (0.94–1.45)*
Riala, 2007*	*Finland*	*1966–2001*	*44*	*22.6*	*1.95 (1.14–2.56)*
Schneider et al. (2011)	Germany	1984–2003	18	7.83	2.30 (1.36–3.48)
Total					1.83 (1.20–2.79) I^2^ = 83 %
Total (misuse)					1.88 (1.78–1.99) I^2^ = 0 %
Total (any use)*					*1.39 (0.83–2.32)* I^2^ = 86 %

⁎ Studies which measured any or unspecified use of tobacco (as opposed to misuse, use disorder, etc.)

**Table 4:** Association between opioid use and suicide.

**Study**	**Country**	**Time**	**N suicides**		**SMR (95 % CI)**
			**Observed**	**Expected**	
**Overall**					
Bjornaas et al. (2008)	Norway	1985–2000	5	0.46	10.87 (3.43–22.48)
Bohnert et al. (2017)	USA	2006–2011	177	63.63	2.78 (2.39–3.21)
Chang et al. (2017)	USA	2006–2014	1	N/A	0.8 (0.01–6.9)
Chang et al. (2017)	Taiwan	2006–2014	27	N/A	18.1 (11.3–24.9)
Crump et al. (2021)	Sweden	2003–2016	206	N/A	9.86 (8.57–11.34)
Lundgren et al. (2022)	Sweden	2003–2017	27	14.19	1.90 (1.25–2.69)
Pan et al. (2014)	Taiwan	1990–2010	75	4.6	16.30 (12.82–20.20)
Padmanathan et al. (2022)	England	1998–2018	46	6.21	7.51 (5.50–10.02)
Park et al. (2019)	South Korea	2002–2013	19	6.97	2.73 (1.64–4.09)
Pavarin et al. (2017)	Italy	1975–2013	47	7.42	6.33 (4.65–8.27)
Pavarin et al. (2019)	Italy	1975–2016	72	13.4	5.37 (4.20–6.69)
Pavarin et al. (2020)	Italy	1975–2016	46	N/A	5.30 (3.97–7.08)
Pierce et al. (2015)	England	2005–2011	351	81.9	4.29 (3.85–4.75)
Veldhuizen and Callaghan, (2014)	USA	1990–2005	231	N/A	6.60 (5.30–7.80)
Total					5.46 (3.66–8.15) I^2^ = 96 %
**Females only**					
Bjornaas et al. (2008)	Norway	1985–2000	3	0.12	25.00 (4.71–61.29)
Bohnert et al. (2017)	USA	2006–2011	9	1.01	8.91 (4.04–15.68)
Crump et al. (2021)	Sweden	2003–2016	61	N/A	15.62 (12.09–20.18)
Padmanathan et al. (2022)	England	1998–2018	9	.64	14.06 (6.38–24.75)
Pan et al. (2014)	Taiwan	1990–2010	9	0.4	22.50 (10.20–39.60)
Pavarin et al. (2017)	Italy	1975–2013	10	0.52	19.23 (9.16–33.00)
Pavarin et al. (2019)	Italy	1975–2016	12	1.0	12.0 (6.17–19.75)
Pavarin et al. (2020)	Italy	1975–2016	N/A	N/A	12.80 (6.66–23.46)
Veldhuizen and Callaghan, (2014)	USA	1990–2005	65	N/A	6.6 (4.60–8.60)
Total					12.63 (8.54–18.68) I^2^ = 69 %
**Males only**					
Bjornaas et al. (2008)	Norway	1985–2000	2	0.35	5.71 (0.54–16.38)
Bohnert et al. (2017)	USA	2006–2011	168	64.55	2.60 (2.22–3.01)
Crump et al. (2021)	Sweden	2003–2016	145	N/A	8.76 (7.41–10.35)
Padmanathan et al. (2022)	England	1998–2018	37	5.49	6.74 (4.74–9.09)
Pan et al. (2014)	Taiwan	1990–2010	66	4.3	15.35 (11.87–19.28)
Pavarin et al. (2017)	Italy	1975–2013	37	6.90	5.36 (3.77–7.23)
Pavarin et al. (2019)	Italy	1975–2016	60	12.4	4.84 (3.69–6.14)
Pavarin et al. (2020)	Italy	1975–2016	N/A	N/A	4.64 (3.36–6.40)
Veldhuizen and Callaghan, (2014)	USA	1990–2005	166	N/A	6.5 (5.00–8.10)
Total					5.58 (3.36–9.24) I^2^ = 96 %

**Table 5:** Association between cannabis use and suicide.

**Study**	**Country**	**Time**	**N suicides**		**SMR (95 % CI)**
			**Observed**	**Expected**	
**Overall**					
Arendt et al. (2013)	Norway		21	N/A	4.8 (2.4–8.9)
Bohnert et al. (2017)	USA	2006–2011	246	94.87	2.58 (2.28–2.93)
Crump et al. (2021)	Sweden	2003–2016	66	N/A	6.19 (4.85–7.89)
Total					3.31 (1.42–7.70) I^2^ = 95 %

**Table 6:** Association between cocaine use and suicide.

**Study**	**Country**	**Time**	**N suicides**		**SMR (95 % CI)**
			**Observed**	**Expected**	
**Overall**					
Bohnert et al. (2017)	USA	2006–2011	231	140.66	1.64 (1.44–1.86)
Crump et al. (2021)	Sweden	2003–2016	7	N/A	5.75 (2.74–12.06)
Pavarin, (2008)	Italy	1989–2004	1	0.02	50.00 (0.02–196.02)
Pavarin and Fioritti, (2018)	Italy	1989–2013	4	0.65	6.15 (1.60–13.66)
Pavarin et al. (2020)	Italy	1975–2016	5	N/A	3.63 (1.51–8.73)
Total					2.02 (0.57–7.20) I^2^ = 84 %

**Table 7:** Association between amphetamine use and suicide.

**Study**	**Country**	**Time**	**N suicides**		**SMR (95 % CI)**
			**Observed**	**Expected**	
**Overall**					
Bohnert et al. (2017)	USA	2006–2011	64	20.2	3.17 (2.44–3.99)
Crump et al. (2021)	Sweden	2003–2016	102	N/A	6.06 (4.98–7.37)
Lee et al. (2021)	Taiwan	2000–2016	745	45.8	16.27 (15.12–17.46)
Total					11.97 (3.13–45.74) I^2^ = 99 %

**Table 8:** Association between mixed/any substance use and suicide.

**Study**	**Country**	**Time**	**N suicides**		**SMR (95 % CI)**
			**Observed**	**Expected**	
Björkenstam et al. (2020)	Sweden	2005–2013	1503	194.44	7.73 (7.34–8.13)
Bøe et al. (2023)**	Norway	2008–2018	99	95.2	1.04 (0.85–1.25)
Bohnert et al. (2017)	USA	2006–2011	1573	648.27	2.43 (2.31–2.55)
Chang et al. (2022)	Taiwan	2000–2016	162	38.67	4.19 (3.57–4.86)
Clapperton et al. (2024)**	Australia	2011–2017	42	25.8	1.63 (1.17–2.16)
Crump et al. (2021)	Sweden	2003–2016	1159	N/A	7.10 (6.65–7.58)
Girardi et al. (2022)	Italy	2008–2018	20	N/A	4.10 (2.36–7.11)
Hesse et al. (2020)	Denmark	2000–2010	163	N/A	7.13 (5.81–8.44)
Høye et al. (2021)	Norway	2009–2015	126	51.69	2.44 (2.03–2.88)
Kauppila et al. (2022)	Mixed Icelandic	1980–2012	10	1.87	5.35 (2.55–9.18)
Kim et al. (2017)	South Korea	2002–2013	172	N/A	6.80 (5.70–7.90)
Lähteenvuo et al. (2021)	Finland	1996–2017	470	285.51	1.65 (1.50–1.80)
Nordentoft et al. (2011)	Denmark	1955–2006	1911	137.13	13.94 (13.32–14.57)
Suominen et al. (2004)	Finland	1997–2002	39	21.71	1.80 (1.28–2.40)
Tidemalm et al. (2008)	Sweden	1973–2003	67	35.8	1.87 (1.45–2.35)
Total					6.83 (3.46–13.47) I^2^ = 99 %

Substance use was associated with higher suicide mortality (SMR: 5.58, 95 % CI: 3.63–8.57, I^2^: 99 %), and results were almost identical when limiting the analysis to studies that examined diagnosed/treated DSM-defined substance use disorder (SUD; SMR 5.62, 3.69–8.55, I^2^: 99 %). In sex-stratified analyses, any substance use was associated with higher rates of suicide mortality for both males and females, but the magnitude of the association was significantly higher for females than males (female SMR: 12.37, 95 % CI: 7.07–21.63, I^2^: 98 %; male SMR: 5.21, 95 % CI: 3.09–8.78, I^2^: 99 %). There was not sufficient information by geographic area or racial/ethnic group for individual substances to conduct analyses by geography or race/ethnicity.

### 3.1 Alcohol

Fifteen studies evaluated the association between alcohol use and suicide (Bøe et al., 2023, Bohnert et al., 2017, Bowden et al., 2018, Chung et al., 2022, Crump et al., 2021, Edwards et al., 2020, Holmstrand et al., 2015, Hung et al., 2015, LeardMann et al., 2013, Mattisson et al., 2011, Pavarin et al., 2020, Ryb et al., 2006, Schneider et al., 2011, Tidemalm et al., 2008, Yi et al., 2016). Alcohol use was associated with significantly increased suicide mortality (pooled SMR: 5.39, 95 % CI: 3.02–9.62, I^2^: 99 %); however, there were differences in magnitude between studies that examined any/unspecified use of alcohol and studies that examined alcohol misuse (i.e., variably defined alcohol misuse, abuse, and alcohol use disorders). Only alcohol misuse was associated with significantly increased suicide mortality rates (misuse SMR: 5.51, 95 % CI: 3.12–9.74, I^2^: 99 %; any/unspecified use SMR: 1.39, 95 % CI: 0.73–2.67, I^2^: 90 %). The SMR point estimates for alcohol misuse were significantly larger for females (SMR: 11.41, 95 % CI: 5.94–21.91, I^2^: 98 %) than for males (SMR: 4.60, 95 % CI: 2.46–8.61, I^2^: 100 %).

### 3.2 Tobacco

Four studies evaluated the association between tobacco use/misuse and suicide (Bohnert et al., 2014, Flensborg-Madsen et al., 2009; Riala, 2007; Schneider et al., 2011). Tobacco use was associated with increased suicide mortality (SMR: 1.83, 95 % CI: 1.20–2.79, I^2^: 83 %). However, findings were similar to alcohol in that only misuse was associated with significantly increased suicide mortality (misuse SMR: 1.88, 95 % CI: 1.78–1.99, I^2^: 0 %; any/unspecified use SMR: 1.39, 95 % CI: 0.83–2.32, I^2^: 86 %). It was not possible to disaggregate these findings by sex due to insufficient studies presenting sex-specific results. Iwasaka and colleagues (2005) reported results from male-only samples and so this study was dropped from analysis; although the strength and direction of its results were consistent with findings from this meta-analysis.

### 3.3 Opioids

Thirteen studies evaluated the association between opioid use and suicide (Bjornaas et al., 2008, Bohnert et al., 2017, Chang et al., 2017, Crump et al., 2021, Lundgren et al., 2022, Pan et al., 2014, Padmanathan et al., 2022, Park et al., 2019, Pavarin et al., 2017, Pavarin et al., 2019, Pavarin et al., 2020, Pierce et al., 2015, Veldhuizen and Callaghan, 2014). Opioid use was associated with significantly increased suicide mortality (SMR: 5.46, 95 % CI: 3.66–8.15, I^2^: 96 %). There were insufficient studies to compare the association between any/unspecified opioid use and opioid misuse/opioid use disorder (OUD). The SMR point estimates for opioid use were significantly larger for females (SMR: 12.63, 95 % CI: 8.54–18.68, I^2^: 69 %) than for males (SMR: 5.58, 95 % CI: 3.36–9.24, I^2^: 96 %).

### 3.4 Cannabis

Three studies evaluated the association between cannabis use and suicide (Arendt et al., 2013, Bohnert et al., 2017, Crump et al., 2021). Cannabis use disorder was associated with significantly increased suicide mortality (SMR 3.31, 95 % CI: 1.42–7.70, I^2^: 95 %). All three studies assessed diagnosed cannabis use disorder, so it was not possible to examine whether lower levels of cannabis use followed the same trend as other licit substances (see Section 3.8 below). It was not possible to disaggregate this category by sex.

### 3.5 Cocaine

Five studies evaluated the association between cocaine use and suicide (Bohnert et al., 2017, Crump et al., 2021, Pavarin, 2008, Pavarin and Fioritti, 2018, Pavarin et al., 2020). Cocaine use was not associated with significantly increased suicide mortality (SMR 2.02, 95 % CI: 0.57–7.20, I^2^: 81 %). It was not possible to disaggregate this category by sex.

### 3.6 Amphetamines

Three studies evaluated the association between amphetamine use and suicide (Bohnert et al., 2017, Crump et al., 2021, Lee et al., 2021). Amphetamine use was associated with significantly increased suicide mortality (SMR 11.97, 95 % CI: 3.13–45.74, I^2^: 99 %). It was not possible to disaggregate this category by sex.

### 3.7 Other/mixed substances

Fifteen studies either did not disaggregate substance use by type or presented results for combined substances in addition to individual substances (Björkenstam et al., 2020, Bøe et al., 2023, Bohnert et al., 2017, Chang et al., 2022, Clapperton et al., 2024, Crump et al., 2021, Girardi et al., 2022, Hesse et al., 2020, Høye et al., 2021, Kauppila et al., 2022, Kim et al., 2017, Lähteenvuo et al., 2021, Nordentoft et al., 2011, Suominen et al., 2004, Tidemalm et al., 2008). In total, the use of other substances was associated with high suicide mortality (SMR: 6.83, 95 % CI: 3.46–13.47, I^2^: 99 %). It was not possible to disaggregate this category by sex.

### 3.8 Licit substances

In light of recent changes in the regulation of substance use (e.g., the legalization of recreational cannabis in some regions), we evaluated the association between common licit substances and suicide. Specifically, we created a category of substance use that combined studies of any or unspecified use of alcohol or tobacco. Any/unspecified use of alcohol or tobacco was not associated with significantly increased suicide mortality (SMR: 1.39, 95 % CI: 0.94–2.06, I^2^: 85 %).

### 3.9 Risk of bias

We evaluated funnel and Doi plots (Doi et al., 2015, Furuya-Kanamori et al., 2018) for evidence of publication bias. The plots that included all studies examined together showed minor asymmetry (LFK index −1.93) which suggests possible, slight publication bias. When substances were examined separately, tobacco and opioids showed no asymmetry, but alcohol, cannabis, cocaine, amphetamines, and other/combined substances showed major asymmetry.

Study quality on the Newcastle-Ottawa Scale (Wells et al., 2009) was generally in the ‘good’ quality range (*M* = 6.00, *SD* = 1.12). We used meta-regression to evaluate the putative role of study quality (i.e., score on the NOS) in moderating results. Meta-regression did not show an association between NOS and effect size (beta coefficient for NOS = 0.12, p = 0.11), suggesting that study quality did not moderate the results.

Results were robust to the exclusion of individual studies for all substances except cocaine. For example, one study of alcohol in Japanese males (Akechi et al., 2006) was the only study to show a significant protective effect of alcohol (or any substance) consumption. However, the exclusion of this study did not substantially alter the pooled SMR for alcohol use in males (4.75, 2.71–8.32, I^2^: 99 %). With cocaine, we tested findings with the removal of two separate studies. One study of cocaine use (Pavarin, 2008) was an outlier, with an SMR of 50 based on a single death in a small sample. The exclusion of this study did not substantially alter either the overall pooled SMR for substance misuse (SMR: 5.54, 95 % CI: 3.60–8.52, I^2^: 99 %) or the pooled SMR for cocaine (SMR: 1.93, 95 % CI: 0.56–6.70, I^2^: 84 %). One study was the only study with a large sample. When excluded (Bohnert et al., 2017, SMR 1.64, 95 % CI: 1.44–1.86) the pooled estimate was significant (SMR 5.50, 95 % CI: 3.29–9.20, I^2^: 0 %).

## 4 Discussion

Over the past two decades, suicide has been recognized globally as a public health challenge with growing inequities across social factors. This updated meta-analysis reaffirms that substance misuse continues to be a risk factor for suicide deaths. This study observed some differences in the associations between substance misuse and suicide compared to those observed by Wilcox and colleagues in 2004. Advancements in clinical practice and drug policies may partially explain this difference. Methodological differences and the inclusion of a higher volume of longitudinal, population-wide studies in this update may also explain differences observed in the strength of the association between substance use and suicide. This study highlights the adverse effects of substance misuse and disorders on risk for suicide mortality, particularly among women.

Alcohol misuse continues to be associated with suicide risk, despite policy changes aimed to limit access to alcohol, the deployment of preventive interventions, and the availability of effective treatments for alcohol use disorder. We observed larger associations between alcohol misuse and suicide than Wilcox and colleagues (2004). This finding may be explained by ongoing barriers to accessing treatment for alcohol misuse. For example, only 1.1 % of those with alcohol use disorder in the US received medication assisted treatment in 2020.(U.S. Department of Health and Human Services, Substance Abuse and Mental Health Services Administration, Center for Behavioral Health Statistics and Quality, 2021) Future implementation science research and practice innovations are needed to promote access to effective treatments for alcohol misuse. Properly addressing alcohol misuse could have downstream effects on suicide mortality.

This study provides novel findings on the association between tobacco use and suicide mortality. Tobacco use was positively associated with suicide death although the magnitude of the overall association with suicide mortality was not as strong as some of the other substances studied. Recent studies have shown an increase in cigarette consumption during and after the COVID-19 pandemic (Carreras et al., 2022, Reitsma et al., 2021). Paired with the growing popularity of e-cigarettes and vaping products (Tehrani et al., 2022), as well as the high rate of co-occurring tobacco misuse and psychiatric disorders that increase suicide risk (Minichino et al., 2013), we recommend researchers continue to investigate the association between tobacco/e-cigarette/vaping, comorbidities, and suicide. Research into the adaptation of tobacco prevention and cessation treatment efforts (Patnode et al., 2021) for people living with mental health conditions is warranted.

This meta-analysis also provides novel finding on the association between cannabis and suicide mortality, in the context of shifting cannabis policy. Cannabis use disorders were associated with significantly elevated rates of suicide mortality. We did not find longitudinal cohort studies interrogating the association between any cannabis use or cannabis misuse with suicide mortality. In light of shifts in cannabis policy that increase access to the substance in the general population, rigorous research is needed to explore whether and how cannabis use broadly increases suicide risk. Research focused on cannabis and polysubstance misuse should be prioritized in the context of changing cannabis policy and shifts in the substance use patterns (Tucker et al., 2021). Existing research suggests that cannabis misuse may increase risk for suicide (Bohnert et al., 2017), although this may be due to increased rates of polysubstance misuse among cannabis users (Arendt et al., 2013, Pavarin and Berardi, 2011).

Opioid use was strongly associated with suicide in this study, although the strength of the association was smaller than that observed by Wilcox and colleagues (2004). In the last 10 years, the rate of overdose deaths has dramatically increased as synthetic opioids have largely replaced heroin (Ciccarone, 2019, National Institute on Drug Abuse, 2023). These findings may reflect advancements in the treatment of opioid use disorders. Medication assisted treatment for OUD appears to be protective against all-cause and suicide mortality (Santo et al., 2021). Alternatively, our findings may reflect the complexity of detecting suicidal intent among overdose decedents that complicates manner of death rulings (Nestadt and Bohnert, 2020). As the majority of data included in this study were collected prior to the influx of fentanyl, future studies should assess the relationship between synthetic opioid misuse and suicide.

Our analysis of the association between psychostimulants and suicide was less clear. Amphetamine misuse was associated with risk for suicide mortality, but cocaine misuse was not associated with suicide risk in pooled analyses. However, all individual studies of cocaine showed a significant association and the removal of a single study from the analysis resulted in a significant pooled risk for suicide mortality and cocaine misuse. The nonsignificant pooled estimate may be a statistical artifact, with exceptionally wide confidence intervals due to pooling a small number of heterogeneous studies with mostly small samples. Assuming a consistent association with psychostimulant use,this finding is alarming because of recent increases in psychostimulant use and in the combined use of stimulants and opioids (Jones et al., 2020). More longitudinal research is needed to evaluate the association between suicide, stimulant and polysubstance misuse, and sequelae of substance misuse including unintentional overdose. Polysubstance misuse is common and increases risk for unintentional overdose (Crummy et al., 2020). While our approach did not parse out the impact of polysubstance use or overdose, our findings are consistent with existing research that suggests that both polysubstance misuse (Martinotti et al., 2009) and unintentional overdose (Olfson et al., 2020) are associated with increased risk for suicidal behaviors. Our study intentionally presents findings for studies that reported out information on mixed/any substance use and suicide. Our findings highlight the significant association between mixed or any substance use and suicide. Further research is recommended to better understand this association, and to better understand if specific substances drive this association to better inform policy and practice. We recommend future researchers present findings disaggregated by substance and use type.

This study partially replicated and extended Wilcox and colleagues’(2004) sex-stratified analyses. Broadly, we found that substance misuse may be more strongly associated with suicide in females, compared to males. This finding may reflect higher rates of comorbidity between behavioral disorders and alcohol misuse among females (Conner et al., 2001). It may also reflect the higher base rate of alcohol misuse in the population of males who are otherwise at low risk for suicide (Bryazka et al., 2022). More research is needed to understand the drivers of disparities in suicide mortality associated with substance misuse and to promote treatment focused on females. We recommend research that evaluates how the acute and chronic misuse of multiple substances can increase risk for suicide, especially following unintentional overdose.

This study aimed to address the association between substance misuse and suicide, disaggregated by socio-demographic characteristics. Few, if any, studies provided sufficient information about race, ethnicity, non-binary gender identity, or sexual orientation of participants. Most articles reported on data from European countries, creating an assumed overrepresentation of White samples in high-income countries in our analysis. The absence of disaggregated data greatly impedes efforts to identify, monitor, and address health inequities. Given well known health inequities for both suicide and substance use, we recommend research with diverse groups, improvement of demographic data capture in health records, capacity building to establish suicide and substance misuse surveillance systems in low- and middle-income countries, the reporting of results disaggregated by demographic factors. As these infrastructure and systems level change are anticipated to take time, we recommend supplementary investigations that specifically assess for potential health inequities.

Our findings likely underestimate the impact of substance use on the probability of suicide due to heterogeneity in the identification of suicide deaths and operationalization of substance misuse. Studies took different approaches to operationalizing suicide mortality, with some studies including probable suicides (e.g., including some deaths of undetermined intent that appeared to be suicide) and including only those deaths ruled to be suicide. This inconsistency reflects the complexity of manner of death rulings in cases of suicide, where it can be difficult to establish decedents’ suicidal intent, and substantial national and international variations when making these determinations. Coroners and medical examiners making manner of death rulings use heterogeneous methods and criteria when investigating potential suicides, creating room for bias associated with decedents’ sex and racial/ethnic background (Ali et al., 2022, Gatov et al., 2018, Rockett et al., 2020). We recommend the development of consistent, operational definitions of suicide to improve data aggregation.

We made several other methodological decisions that may result in underestimations of suicide for people who misuse substances, especially for those who may be especially vulnerable to suicide. We restricted analyses to longitudinal cohort studies that avoided high rates of attrition, which necessarily excluded groups where dropouts may have reflected more severe conditions or worse longer-term outcomes. We did not include studies involving cohorts of persons with primary psychiatric disorders, neurodevelopmental differences, or suicide attempt histories where many participants may have suffered comorbid substance use disorders. Existing research suggest that people with co-occurring psychiatric and substance use disorders (Najt et al., 2011), people who are on the autism spectrum and misuse substances (Lai et al., 2023), and people who have attempted suicide and misuse substances (Edwards et al., 2024) are particularly vulnerable to suicidal thoughts and behaviors. We pooled studies that may have different inclusion/exclusion criteria between them, which may have contributed to the high degree of heterogeneity we observed. A further limitation of this study was the lack of consistency in defining substance use exposures. The studies included had different ways of defining substance use, misuse, and substance use disorders and in quantifying the duration of substance misuse. Given these limitations, we could not discern whether or not there was a dose dependent association between substance use and suicide mortality, or whether there is an association between the social consequences of a substance use disorder and suicide mortality (Choi et al., 2018, Conner et al., 2019, Yuodelis-Flores and Ries, 2015). Future research should operationalize substance use exposures consistently, address a wider range of substances, and address the interaction between substance misuse and other suicide risk factors.

This systematic review also revealed shifts in statistical approaches used in longitudinal cohort studies. We developed this review to build upon meta-analyses conducted by Harris and Barraclough (1997) and Wilcox and colleagues (2004). In line with this prior work, we estimated pooled SMRs that describe the association between substance misuse and suicide and excluded studies that did not provide sufficient information from which to calculate SMR (i.e., the observed and expected number of deaths, *n* of the exposed population and general population control groups). Ultimately, we excluded 67 studies that did not provide sufficient data for extraction and 19 studies that did not provide data for the general population control group. Frequently, these studies used alternative statistics (e.g., hazard ratios) to describe the association between substance misuse and suicide. Given this shift in statistical approaches to longitudinal cohort studies over time, future systematic reviews and meta-analyses may benefit from selecting alterative data extraction and meta-analytic approaches that captures findings from studies that take this alternative approach to quantifying risk.

## 5 Conclusions

Substance use disorders remain an important risk factor for suicide mortality. A challenge within the field of psychiatry has been trying to find methods to accurately predict suicide risk in order to prevent it. For example, several studies have demonstrated that suicide risk assessment scales are often not accurate or backed by sufficient evidence. The findings in this study demonstrate markedly increased risk of suicide death in those with substance misuse and substance use disorders. Without evidence-backed formal suicide assessment tools available, the treatment of substance misuse and substance use disorders are a target in the clinical setting to reduce suicide mortality. The findings in this study should be considered in the context of growing rates of substance misuse and suicide globally (Ilic and Ilic, 2022, U.S, 2020, United Nations Office on Drugs and Crime, 2022), as well as significant changes in clinical practice and drug policy. Additionally, targeting substance use at the public health, and policy level has the potential to reduce suicide rates. To make a substantial impact on the population rate of suicide, it is necessary to prioritize the primary prevention of substance misuse and co-occurring psychiatric disorders that are linked to suicide. Additionally, advancements in implementation research and practice are needed to promote access to equitable and effective interventions for substance misuse.

## Funding

Dr. Athey was supported by Grant Number T32MH014592 from the 10.13039/100000025NIMH at the time of data collection for this study. Dr. Nestadt was supported by Grant Number YIG-0-093-18 from the American Foundation for Suicide Prevention at the time of manuscript preparation. He is currently supported by National Institutes of Drug Abuse under award number K23DA055693. Dr. Wilcox is supported by Grant Number TR34MH121639 and Grant Number R01MH122214 from NIMH. Drs. Athey, Nestadt, and Wilcox are supported by Grant Number PRG-0–011–20 from the American Foundation for Suicide Prevention.

## CRediT authorship contribution statement

**Geoffrey Kahn:** Writing – review & editing, Writing – original draft, Visualization, Software, Formal analysis, Data curation, Conceptualization. **Jaimie Shaff:** Writing – review & editing, Writing – original draft, Validation, Supervision, Project administration, Methodology, Investigation, Formal analysis, Data curation, Conceptualization. **Alison Athey:** Writing – review & editing, Writing – original draft, Validation, Supervision, Software, Project administration, Methodology, Investigation, Data curation, Conceptualization. **Holly C. Wilcox:** Writing – review & editing, Writing – original draft, Supervision, Resources, Methodology, Conceptualization. **Paul S. Nestadt:** Writing – review & editing, Writing – original draft, Conceptualization, Data curation. **Aubrey DeVinney:** Writing – review & editing, Writing – original draft, Data curation. **Holly Sawyer:** Writing – original draft, Data curation. **Taylor C. Ryan:** Writing – review & editing, Writing – original draft, Data curation, Formal analysis. **Kathryn Brodie:** Writing – review & editing, Writing – original draft, Data curation.

## Declaration of Competing Interest

Nothing Declared.
