# Supplementary material for: Association of substance use with suicide mortality: An updated systematic review and meta-analysis
Source: Drug Alcohol Depend Rep. 2024 Dec 13;14:100310. doi: 10.1016/j.dadr.2024.100310 (PMC11741031; doi:10.1016/j.dadr.2024.100310)
Supplement: Supplementary file 1 — Supplementary material [file mmc1.docx]

**Supplemental Table 1. Search Strategy**

| **Pub Med** | **Search Terms** |
| --- | --- |
| 1 | ("Suicide"[MESH] OR suicid*[tw]) |
| 2 | ("Substance-Related Disorders"[MESH] OR "Alcoholism"[MESH] OR "Alcohol-Related Disorders"[MESH] OR "Alcohol Drinking"[MESH] OR substance*[tw] OR drug*[tw] OR opioid*[tw] OR opiate* [tw] OR alcohol*[tw] OR cocaine*[tw] OR marijuana*[tw] OR inhalant*[tw] OR phencyclidine*[tw] OR amphetamine*[tw] benzodiazepine* [tw] OR morphine*[tw] OR heroin*[tw] OR fentanyl*[tw] OR oxycodone*[tw] OR codeine*[tw] OR hydrocodone*[tw] OR methadone*[tw] OR MDMA[tw] OR methamphetamine*[tw]) |
| 3 | ("Mortality"[MESH] OR "Death"[MESH] OR "Fatal Outcome"[MESH] mortality [tw] OR fatal*[tw] OR death*[tw]) |
| 4 | #1 AND #2 AND #3 |
| 5 | (Animals[mesh] NOT humans[mesh]) |
| 6 | #4 NOT #5* |
| **EMBASE** | **Search Terms** |
| 1 | ('suicide'/exp) OR (suicid*):ti,ab |
| 2 | ('drug dependence'/exp) OR ('substance use'/exp) OR ('drug abuse'/exp) OR (substance* OR drug* OR opioid* OR opiate* OR alcohol* OR cocaine* OR marijuana* OR inhalant* OR phencyclidine* OR amphetamine* OR benzodiazepine* OR morphine* OR heroin* OR fentanyl* OR oxycodone* OR codeine* OR hydrocodone* OR methadone* OR mdma OR methamphetamine*):ti,ab |
| 3 | ('mortality'/exp) OR ('death'/exp) OR (mortality OR death* OR fatal*):ti,ab |
| 4 | #1 AND #2 AND #3 |
| 5 | animals'/exp NOT 'humans'/exp |
| 6 | #4 NOT #5 |
| 7 | ([conference abstract]/lim OR [conference paper]/lim OR [editorial]/lim OR [erratum]/lim OR [letter]/lim OR [note]/lim OR [short survey]/lim) |
| 8 | #6 NOT #7* |
| **CINAHL** | **Search Terms** |
| 1 | (MH "Suicide+") OR TI suicid* OR AB suicid* |
| 2 | (MH "substance use disorders+") OR (MH "alcohol drinking+") OR TI substance* OR TI drug* OR TI opioid* OR TI opiate* OR TI alcohol* OR TI cocaine* OR TI marijuana* OR TI inhalant* OR TI phencyclidine* OR TI amphetamine* benzodiazepine* OR TI morphine* OR TI heroin* OR TI fentanyl* OR TI oxycodone* OR TI codeine* OR TI hydrocodone* OR TI methadone* OR TI MDMA OR TI methamphetamine* OR AB substance* OR AB drug* OR AB opioid* OR AB opiate* OR AB alcohol* OR AB cocaine* OR AB marijuana* OR AB inhalant* OR AB phencyclidine* OR AB amphetamine* benzodiazepine* OR AB morphine* OR AB heroin* OR AB fentanyl* OR AB oxycodone* OR AB codeine* OR AB hydrocodone* OR AB methadone* OR AB MDMA OR AB methamphetamine* |
| 3 | (MH "mortality+") OR TI mortality OR AB mortality OR (MH "death+") OR TI death* OR AB death* OR TI fatal* OR AB fatal* |
| 4 | S1 AND S2 AND S3 |
| 5 | (MH "animals") NOT ("MH "humans") |
| 6 | S4 NOT S5* |
|  |  |
| **PsycINFO** | **Search Terms** |
| 1 | (MH "Suicide+") OR TI suicid* OR AB suicid* |
| 2 | (MH "Substance Use Disorder+") OR (MH "drug usage+") OR TI substance* OR TI drug* OR TI opioid* OR TI opiate* OR TI alcohol* OR TI cocaine* OR TI marijuana* OR TI inhalant* OR TI phencyclidine* OR TI amphetamine* benzodiazepine* OR TI morphine* OR TI heroin* OR TI fentanyl* OR TI oxycodone* OR TI codeine* OR TI hydrocodone* OR TI methadone* OR TI MDMA OR TI methamphetamine* OR AB substance* OR AB drug* OR AB opioid* OR AB opiate* OR AB alcohol* OR AB cocaine* OR AB marijuana* OR AB inhalant* OR AB phencyclidine* OR AB amphetamine* benzodiazepine* OR AB morphine* OR AB heroin* OR AB fentanyl* OR AB oxycodone* OR AB codeine* OR AB hydrocodone* OR AB methadone* OR AB MDMA OR AB methamphetamine* |
| 3 | (MH "Death and Dying+") OR TI mortality OR AB mortality OR TI death* OR AB death* OR TI fatal* OR AB fatal* |
| 4 | S1 AND S2 AND S3 |
| 5 | (MH "Animals") NOT (MH "Humans") |
| 6 | S4 NOT S5* |
|  |  |
| **Cochrane** | **Search Terms** |
| 1 | ([mh Suicide] OR suicid*):ti,ab |
| 2 | ([mh "Substance-Related Disorders"] OR [mh "Alcoholism"] OR [mh "Alcohol-Related Disorders"] OR [mh "Alcohol Drinking"] OR substance* OR drug* OR opioid* OR opiate* OR alcohol* OR cocaine* OR marijuana* OR inhalant* OR phencyclidine* OR amphetamine* benzodiazepine* OR morphine* OR heroin* OR fentanyl* OR oxycodone* OR codeine* OR hydrocodone* OR methadone* OR MDMA OR methamphetamine*):ti,ab |
| 3 | ([mh Mortality] OR [mh Death] OR [mh "Fatal Outcome"] OR mortality OR fatal* OR death*):ti,ab |
| 4 | #1 AND #2 AND #3 |
| 5 | ("Animals"[MESH]) NOT ("Humans" [MESH]) |
| 6 | #4 NOT #5* |

* limit by date and language

|  |
| --- |

**Supplemental Table 2. Data Extraction Form**

| **Study Characteristics** | |
| --- | --- |
| Study name (Author Year) |  |
| Study Title |  |
| Corresponding Author Email |  |
| Conflicts of Interest  Did the authors report conflicts of interest? | Yes |
|  | No |
|  | Other: |
| Country where study was conducted |  |
| Study Design | Prospective cohort study |
|  | Retrospective cohort study |
| Describe follow-up method |  |
| Average Years of Follow-Up |  |
| Start of Follow-Up Period (year) |  |
| End of Follow-up Period (year) |  |
| Recruitment Method | Geographical catchment area |
|  | Data registry |
|  | Clinic patients |
|  | Random Digit Dialing |
|  | School |
|  | Other |
| Total Sample N |  |
| **Study Population** | |
| Average Age at Recruitment |  |
| Developmental Period at Recruitment | Childhood (0-12) |
|  | Adolescence (13-17) |
|  | Young Adult (18-39) |
|  | Adult (40-64) |
|  | Elder (65+) |
| Racial Ethnic Composition of the Sample |  |
| White |  |
| N |  |
| % |  |
| N Suicides |  |
| % Suicides |  |
| Black |  |
| N |  |
| % |  |
| N Suicides |  |
| % Suicides |  |
| Asian/Pacific Islander |  |
| N |  |
| % |  |
| N Suicides |  |
| % Suicides |  |
| Latinx |  |
| N |  |
| % |  |
| N Suicides |  |
| % Suicides |  |
| Native/Indigenous |  |
| N |  |
| % |  |
| N Suicides |  |
| % Suicides |  |
| More than one race/ethnicity |  |
| N |  |
| % |  |
| N Suicides |  |
| % Suicides |  |
| Other |  |
| N |  |
| % |  |
| N Suicides |  |
| % Suicides |  |
| Unknown |  |
| N |  |
| % |  |
| N Suicides |  |
| % Suicides |  |
| Not Provided |  |
| N |  |
| % |  |
| N Suicides |  |
| % Suicides |  |
| Gender Composition of Sample |  |
| Male |  |
| N |  |
| % |  |
| N Suicides |  |
| % Suicides |  |
| Female |  |
| N |  |
| % |  |
| N Suicides |  |
| % Suicides |  |
| Transgender |  |
| N |  |
| % |  |
| N Suicides |  |
| % Suicides |  |
| Other |  |
| N |  |
| % |  |
| N Suicides |  |
| % Suicides |  |
| Unknown |  |
| N |  |
| % |  |
| N Suicides |  |
| % Suicides |  |
| Not Provided |  |
| N |  |
| % |  |
| N Suicides |  |
| % Suicides |  |
| Sexual Orientation of the Sample |  |
| Heterosexual |  |
| N |  |
| % |  |
| N Suicides |  |
| % Suicides |  |
| Bisexual |  |
| N |  |
| % |  |
| N Suicides |  |
| % Suicides |  |
| Homosexual |  |
| N |  |
| % |  |
| N Suicides |  |
| % Suicides |  |
| Other |  |
| N |  |
| % |  |
| N Suicides |  |
| % Suicides |  |
| Unknown |  |
| N |  |
| % |  |
| N Suicides |  |
| % Suicides |  |
| **Methods** | |
| Substances Assessed | Alcohol |
|  | Tobacco |
|  | Cannabis |
|  | Cocaine |
|  | Amphetamines |
|  | Methamphetamine |
|  | Benzodiazepines |
|  | MDMA |
|  | Opioids: heroin, codeine, morphine |
|  | Synthetic opioids (e.g., fentanyl) |
|  | Mixed/Any Opioids |
|  | Inhalants |
|  | PCP |
|  | Mixed |
|  | Other: |
| Method of use | Smoked |
|  | Vaped |
|  | Inhaled (snort/sniff) |
|  | Injected |
|  | Ingested (eat/drink) |
|  | Not specified |
|  | Other: |
| Type(s) of Substance Use | Any use |
|  | Misuse |
|  | Diagnosed Use Disorder |
|  | Overdose |
|  | Treatment |
| How was substance use assessed? | Self-report |
|  | Interview |
|  | Medical Records |
|  | Service Utilization |
|  | Toxicology from bio sample |
|  | Other: |
| Outcomes assessed | Suicides |
|  | Probable suicides |
|  | Deaths of undetermined intent (do not extract data) |
|  | Accidental Deaths (do not extract data) |
|  | Homicides (do not extract data) |
|  | All-cause mortality (do not extract data) |
| **Observed and Expected Suicide Data** | |
| *Any substance use* |  |
| Alcohol |  |
| Subsample N |  |
| Observed Suicides |  |
| Expected Suicides |  |
| SMR |  |
| Male N |  |
| Male Observed Suicides |  |
| Male Expected Suicides |  |
| Male SMR |  |
| Female N |  |
| Female Observed Suicides |  |
| Female Expected Suicides |  |
| Female SMR |  |
| Tobacco |  |
| Subsample N |  |
| Observed Suicides |  |
| Expected Suicides |  |
| SMR |  |
| Male N |  |
| Male Observed Suicides |  |
| Male Expected Suicides |  |
| Male SMR |  |
| Female N |  |
| Female Observed Suicides |  |
| Female Expected Suicides |  |
| Female SMR |  |
| Cannabis |  |
| Subsample N |  |
| Observed Suicides |  |
| Expected Suicides |  |
| SMR |  |
| Male N |  |
| Male Observed Suicides |  |
| Male Expected Suicides |  |
| Male SMR |  |
| Female N |  |
| Female Observed Suicides |  |
| Female Expected Suicides |  |
| Female SMR |  |
| Cocaine |  |
| Subsample N |  |
| Observed Suicides |  |
| Expected Suicides |  |
| SMR |  |
| Male N |  |
| Male Observed Suicides |  |
| Male Expected Suicides |  |
| Male SMR |  |
| Female N |  |
| Female Observed Suicides |  |
| Female Expected Suicides |  |
| Female SMR |  |
| Amphetamines |  |
| Subsample N |  |
| Observed Suicides |  |
| Expected Suicides |  |
| SMR |  |
| Male N |  |
| Male Observed Suicides |  |
| Male Expected Suicides |  |
| Male SMR |  |
| Female N |  |
| Female Observed Suicides |  |
| Female Expected Suicides |  |
| Female SMR |  |
| Methamphetamines |  |
| Subsample N |  |
| Observed Suicides |  |
| Expected Suicides |  |
| SMR |  |
| Male N |  |
| Male Observed Suicides |  |
| Male Expected Suicides |  |
| Male SMR |  |
| Female N |  |
| Female Observed Suicides |  |
| Female Expected Suicides |  |
| Female SMR |  |
| Benzodiazepines |  |
| Subsample N |  |
| Observed Suicides |  |
| Expected Suicides |  |
| SMR |  |
| Male N |  |
| Male Observed Suicides |  |
| Male Expected Suicides |  |
| Male SMR |  |
| Female N |  |
| Female Observed Suicides |  |
| Female Expected Suicides |  |
| Female SMR |  |
| MDMA |  |
| Subsample N |  |
| Observed Suicides |  |
| Expected Suicides |  |
| SMR |  |
| Male N |  |
| Male Observed Suicides |  |
| Male Expected Suicides |  |
| Male SMR |  |
| Female N |  |
| Female Observed Suicides |  |
| Female Expected Suicides |  |
| Female SMR |  |
| Opioids: heroin, codeine, morphine |  |
| Subsample N |  |
| Observed Suicides |  |
| Expected Suicides |  |
| SMR |  |
| Male N |  |
| Male Observed Suicides |  |
| Male Expected Suicides |  |
| Male SMR |  |
| Female N |  |
| Female Observed Suicides |  |
| Female Expected Suicides |  |
| Female SMR |  |
| Synthetic Opioids |  |
| Subsample N |  |
| Observed Suicides |  |
| Expected Suicides |  |
| SMR |  |
| Male N |  |
| Male Observed Suicides |  |
| Male Expected Suicides |  |
| Male SMR |  |
| Female N |  |
| Female Observed Suicides |  |
| Female Expected Suicides |  |
| Female SMR |  |
| Mixed/Any Opioids |  |
| Subsample N |  |
| Observed Suicides |  |
| Expected Suicides |  |
| SMR |  |
| Male N |  |
| Male Observed Suicides |  |
| Male Expected Suicides |  |
| Male SMR |  |
| Female N |  |
| Female Observed Suicides |  |
| Female Expected Suicides |  |
| Female SMR |  |
| Inhalants |  |
| Subsample N |  |
| Observed Suicides |  |
| Expected Suicides |  |
| SMR |  |
| Male N |  |
| Male Observed Suicides |  |
| Male Expected Suicides |  |
| Male SMR |  |
| Female N |  |
| Female Observed Suicides |  |
| Female Expected Suicides |  |
| Female SMR |  |
| PCP |  |
| Subsample N |  |
| Observed Suicides |  |
| Expected Suicides |  |
| SMR |  |
| Male N |  |
| Male Observed Suicides |  |
| Male Expected Suicides |  |
| Male SMR |  |
| Female N |  |
| Female Observed Suicides |  |
| Female Expected Suicides |  |
| Female SMR |  |
| Mixed Substances |  |
| Subsample N |  |
| Observed Suicides |  |
| Expected Suicides |  |
| SMR |  |
| Male N |  |
| Male Observed Suicides |  |
| Male Expected Suicides |  |
| Male SMR |  |
| Female N |  |
| Female Observed Suicides |  |
| Female Expected Suicides |  |
| Female SMR |  |
| IV Drugs |  |
| Subsample N |  |
| Observed Suicides |  |
| Expected Suicides |  |
| SMR |  |
| Male N |  |
| Male Observed Suicides |  |
| Male Expected Suicides |  |
| Male SMR |  |
| Female N |  |
| Female Observed Suicides |  |
| Female Expected Suicides |  |
| Female SMR |  |
| Other: |  |
| Subsample N |  |
| Observed Suicides |  |
| Expected Suicides |  |
| SMR |  |
| Male N |  |
| Male Observed Suicides |  |
| Male Expected Suicides |  |
| Male SMR |  |
| Female N |  |
| Female Observed Suicides |  |
| Female Expected Suicides |  |
| Female SMR |  |
| Any Substance Use |  |
| Subsample N |  |
| Observed Suicides |  |
| Expected Suicides |  |
| SMR |  |
| Male N |  |
| Male Observed Suicides |  |
| Male Expected Suicides |  |
| Male SMR |  |
| Female N |  |
| Female Observed Suicides |  |
| Female Expected Suicides |  |
| Female SMR |  |
| Non-exposed Control Group |  |
| Subsample N |  |
| Observed Suicides |  |
| Expected Suicides |  |
| SMR |  |
| Male N |  |
| Male Observed Suicides |  |
| Male Expected Suicides |  |
| Male SMR |  |
| Female N |  |
| Female Observed Suicides |  |
| Female Expected Suicides |  |
| Female SMR |  |
| *Substance Misuse* |  |
| Alcohol |  |
| Subsample N |  |
| Observed Suicides |  |
| Expected Suicides |  |
| SMR |  |
| Male N |  |
| Male Observed Suicides |  |
| Male Expected Suicides |  |
| Male SMR |  |
| Female N |  |
| Female Observed Suicides |  |
| Female Expected Suicides |  |
| Female SMR |  |
| Tobacco |  |
| Subsample N |  |
| Observed Suicides |  |
| Expected Suicides |  |
| SMR |  |
| Male N |  |
| Male Observed Suicides |  |
| Male Expected Suicides |  |
| Male SMR |  |
| Female N |  |
| Female Observed Suicides |  |
| Female Expected Suicides |  |
| Female SMR |  |
| Cannabis |  |
| Subsample N |  |
| Observed Suicides |  |
| Expected Suicides |  |
| SMR |  |
| Male N |  |
| Male Observed Suicides |  |
| Male Expected Suicides |  |
| Male SMR |  |
| Female N |  |
| Female Observed Suicides |  |
| Female Expected Suicides |  |
| Female SMR |  |
| Cocaine |  |
| Subsample N |  |
| Observed Suicides |  |
| Expected Suicides |  |
| SMR |  |
| Male N |  |
| Male Observed Suicides |  |
| Male Expected Suicides |  |
| Male SMR |  |
| Female N |  |
| Female Observed Suicides |  |
| Female Expected Suicides |  |
| Female SMR |  |
| Amphetamines |  |
| Subsample N |  |
| Observed Suicides |  |
| Expected Suicides |  |
| SMR |  |
| Male N |  |
| Male Observed Suicides |  |
| Male Expected Suicides |  |
| Male SMR |  |
| Female N |  |
| Female Observed Suicides |  |
| Female Expected Suicides |  |
| Female SMR |  |
| Methamphetamines |  |
| Subsample N |  |
| Observed Suicides |  |
| Expected Suicides |  |
| SMR |  |
| Male N |  |
| Male Observed Suicides |  |
| Male Expected Suicides |  |
| Male SMR |  |
| Female N |  |
| Female Observed Suicides |  |
| Female Expected Suicides |  |
| Female SMR |  |
| Benzodiazepines |  |
| Subsample N |  |
| Observed Suicides |  |
| Expected Suicides |  |
| SMR |  |
| Male N |  |
| Male Observed Suicides |  |
| Male Expected Suicides |  |
| Male SMR |  |
| Female N |  |
| Female Observed Suicides |  |
| Female Expected Suicides |  |
| Female SMR |  |
| MDMA |  |
| Subsample N |  |
| Observed Suicides |  |
| Expected Suicides |  |
| SMR |  |
| Male N |  |
| Male Observed Suicides |  |
| Male Expected Suicides |  |
| Male SMR |  |
| Female N |  |
| Female Observed Suicides |  |
| Female Expected Suicides |  |
| Female SMR |  |
| Opioids: heroin, codeine, morphine |  |
| Subsample N |  |
| Observed Suicides |  |
| Expected Suicides |  |
| SMR |  |
| Male N |  |
| Male Observed Suicides |  |
| Male Expected Suicides |  |
| Male SMR |  |
| Female N |  |
| Female Observed Suicides |  |
| Female Expected Suicides |  |
| Female SMR |  |
| Synthetic Opioids |  |
| Subsample N |  |
| Observed Suicides |  |
| Expected Suicides |  |
| SMR |  |
| Male N |  |
| Male Observed Suicides |  |
| Male Expected Suicides |  |
| Male SMR |  |
| Female N |  |
| Female Observed Suicides |  |
| Female Expected Suicides |  |
| Female SMR |  |
| Mixed/Any Opioids |  |
| Subsample N |  |
| Observed Suicides |  |
| Expected Suicides |  |
| SMR |  |
| Male N |  |
| Male Observed Suicides |  |
| Male Expected Suicides |  |
| Male SMR |  |
| Female N |  |
| Female Observed Suicides |  |
| Female Expected Suicides |  |
| Female SMR |  |
| Inhalants |  |
| Subsample N |  |
| Observed Suicides |  |
| Expected Suicides |  |
| SMR |  |
| Male N |  |
| Male Observed Suicides |  |
| Male Expected Suicides |  |
| Male SMR |  |
| Female N |  |
| Female Observed Suicides |  |
| Female Expected Suicides |  |
| Female SMR |  |
| PCP |  |
| Subsample N |  |
| Observed Suicides |  |
| Expected Suicides |  |
| SMR |  |
| Male N |  |
| Male Observed Suicides |  |
| Male Expected Suicides |  |
| Male SMR |  |
| Female N |  |
| Female Observed Suicides |  |
| Female Expected Suicides |  |
| Female SMR |  |
| Mixed Substances |  |
| Subsample N |  |
| Observed Suicides |  |
| Expected Suicides |  |
| SMR |  |
| Male N |  |
| Male Observed Suicides |  |
| Male Expected Suicides |  |
| Male SMR |  |
| Female N |  |
| Female Observed Suicides |  |
| Female Expected Suicides |  |
| Female SMR |  |
| IV Drugs |  |
| Subsample N |  |
| Observed Suicides |  |
| Expected Suicides |  |
| SMR |  |
| Male N |  |
| Male Observed Suicides |  |
| Male Expected Suicides |  |
| Male SMR |  |
| Female N |  |
| Female Observed Suicides |  |
| Female Expected Suicides |  |
| Female SMR |  |
| Other: |  |
| Subsample N |  |
| Observed Suicides |  |
| Expected Suicides |  |
| SMR |  |
| Male N |  |
| Male Observed Suicides |  |
| Male Expected Suicides |  |
| Male SMR |  |
| Female N |  |
| Female Observed Suicides |  |
| Female Expected Suicides |  |
| Female SMR |  |
| Any Substance Use |  |
| Subsample N |  |
| Observed Suicides |  |
| Expected Suicides |  |
| SMR |  |
| Male N |  |
| Male Observed Suicides |  |
| Male Expected Suicides |  |
| Male SMR |  |
| Female N |  |
| Female Observed Suicides |  |
| Female Expected Suicides |  |
| Female SMR |  |
| Non-exposed Control Group |  |
| Subsample N |  |
| Observed Suicides |  |
| Expected Suicides |  |
| SMR |  |
| Male N |  |
| Male Observed Suicides |  |
| Male Expected Suicides |  |
| Male SMR |  |
| Female N |  |
| Female Observed Suicides |  |
| Female Expected Suicides |  |
| Female SMR |  |
| *Substance Use Disorder* |  |
| Alcohol |  |
| Subsample N |  |
| Observed Suicides |  |
| Expected Suicides |  |
| SMR |  |
| Male N |  |
| Male Observed Suicides |  |
| Male Expected Suicides |  |
| Male SMR |  |
| Female N |  |
| Female Observed Suicides |  |
| Female Expected Suicides |  |
| Female SMR |  |
| Tobacco |  |
| Subsample N |  |
| Observed Suicides |  |
| Expected Suicides |  |
| SMR |  |
| Male N |  |
| Male Observed Suicides |  |
| Male Expected Suicides |  |
| Male SMR |  |
| Female N |  |
| Female Observed Suicides |  |
| Female Expected Suicides |  |
| Female SMR |  |
| Cannabis |  |
| Subsample N |  |
| Observed Suicides |  |
| Expected Suicides |  |
| SMR |  |
| Male N |  |
| Male Observed Suicides |  |
| Male Expected Suicides |  |
| Male SMR |  |
| Female N |  |
| Female Observed Suicides |  |
| Female Expected Suicides |  |
| Female SMR |  |
| Cocaine |  |
| Subsample N |  |
| Observed Suicides |  |
| Expected Suicides |  |
| SMR |  |
| Male N |  |
| Male Observed Suicides |  |
| Male Expected Suicides |  |
| Male SMR |  |
| Female N |  |
| Female Observed Suicides |  |
| Female Expected Suicides |  |
| Female SMR |  |
| Amphetamines |  |
| Subsample N |  |
| Observed Suicides |  |
| Expected Suicides |  |
| SMR |  |
| Male N |  |
| Male Observed Suicides |  |
| Male Expected Suicides |  |
| Male SMR |  |
| Female N |  |
| Female Observed Suicides |  |
| Female Expected Suicides |  |
| Female SMR |  |
| Methamphetamines |  |
| Subsample N |  |
| Observed Suicides |  |
| Expected Suicides |  |
| SMR |  |
| Male N |  |
| Male Observed Suicides |  |
| Male Expected Suicides |  |
| Male SMR |  |
| Female N |  |
| Female Observed Suicides |  |
| Female Expected Suicides |  |
| Female SMR |  |
| Benzodiazepines |  |
| Subsample N |  |
| Observed Suicides |  |
| Expected Suicides |  |
| SMR |  |
| Male N |  |
| Male Observed Suicides |  |
| Male Expected Suicides |  |
| Male SMR |  |
| Female N |  |
| Female Observed Suicides |  |
| Female Expected Suicides |  |
| Female SMR |  |
| MDMA |  |
| Subsample N |  |
| Observed Suicides |  |
| Expected Suicides |  |
| SMR |  |
| Male N |  |
| Male Observed Suicides |  |
| Male Expected Suicides |  |
| Male SMR |  |
| Female N |  |
| Female Observed Suicides |  |
| Female Expected Suicides |  |
| Female SMR |  |
| Opioids: heroin, codeine, morphine |  |
| Subsample N |  |
| Observed Suicides |  |
| Expected Suicides |  |
| SMR |  |
| Male N |  |
| Male Observed Suicides |  |
| Male Expected Suicides |  |
| Male SMR |  |
| Female N |  |
| Female Observed Suicides |  |
| Female Expected Suicides |  |
| Female SMR |  |
| Synthetic Opioids |  |
| Subsample N |  |
| Observed Suicides |  |
| Expected Suicides |  |
| SMR |  |
| Male N |  |
| Male Observed Suicides |  |
| Male Expected Suicides |  |
| Male SMR |  |
| Female N |  |
| Female Observed Suicides |  |
| Female Expected Suicides |  |
| Female SMR |  |
| Mixed/Any Opioids |  |
| Subsample N |  |
| Observed Suicides |  |
| Expected Suicides |  |
| SMR |  |
| Male N |  |
| Male Observed Suicides |  |
| Male Expected Suicides |  |
| Male SMR |  |
| Female N |  |
| Female Observed Suicides |  |
| Female Expected Suicides |  |
| Female SMR |  |
| Inhalants |  |
| Subsample N |  |
| Observed Suicides |  |
| Expected Suicides |  |
| SMR |  |
| Male N |  |
| Male Observed Suicides |  |
| Male Expected Suicides |  |
| Male SMR |  |
| Female N |  |
| Female Observed Suicides |  |
| Female Expected Suicides |  |
| Female SMR |  |
| PCP |  |
| Subsample N |  |
| Observed Suicides |  |
| Expected Suicides |  |
| SMR |  |
| Male N |  |
| Male Observed Suicides |  |
| Male Expected Suicides |  |
| Male SMR |  |
| Female N |  |
| Female Observed Suicides |  |
| Female Expected Suicides |  |
| Female SMR |  |
| Mixed Substances |  |
| Subsample N |  |
| Observed Suicides |  |
| Expected Suicides |  |
| SMR |  |
| Male N |  |
| Male Observed Suicides |  |
| Male Expected Suicides |  |
| Male SMR |  |
| Female N |  |
| Female Observed Suicides |  |
| Female Expected Suicides |  |
| Female SMR |  |
| IV Drugs |  |
| Subsample N |  |
| Observed Suicides |  |
| Expected Suicides |  |
| SMR |  |
| Male N |  |
| Male Observed Suicides |  |
| Male Expected Suicides |  |
| Male SMR |  |
| Female N |  |
| Female Observed Suicides |  |
| Female Expected Suicides |  |
| Female SMR |  |
| Other: |  |
| Subsample N |  |
| Observed Suicides |  |
| Expected Suicides |  |
| SMR |  |
| Male N |  |
| Male Observed Suicides |  |
| Male Expected Suicides |  |
| Male SMR |  |
| Female N |  |
| Female Observed Suicides |  |
| Female Expected Suicides |  |
| Female SMR |  |
| Any Substance Use |  |
| Subsample N |  |
| Observed Suicides |  |
| Expected Suicides |  |
| SMR |  |
| Male N |  |
| Male Observed Suicides |  |
| Male Expected Suicides |  |
| Male SMR |  |
| Female N |  |
| Female Observed Suicides |  |
| Female Expected Suicides |  |
| Female SMR |  |
| Non-exposed Control Group |  |
| Subsample N |  |
| Observed Suicides |  |
| Expected Suicides |  |
| SMR |  |
| Male N |  |
| Male Observed Suicides |  |
| Male Expected Suicides |  |
| Male SMR |  |
| Female N |  |
| Female Observed Suicides |  |
| Female Expected Suicides |  |
| Female SMR |  |
| *Overdose* |  |
| Alcohol |  |
| Subsample N |  |
| Observed Suicides |  |
| Expected Suicides |  |
| SMR |  |
| Male N |  |
| Male Observed Suicides |  |
| Male Expected Suicides |  |
| Male SMR |  |
| Female N |  |
| Female Observed Suicides |  |
| Female Expected Suicides |  |
| Female SMR |  |
| Tobacco |  |
| Subsample N |  |
| Observed Suicides |  |
| Expected Suicides |  |
| SMR |  |
| Male N |  |
| Male Observed Suicides |  |
| Male Expected Suicides |  |
| Male SMR |  |
| Female N |  |
| Female Observed Suicides |  |
| Female Expected Suicides |  |
| Female SMR |  |
| Cannabis |  |
| Subsample N |  |
| Observed Suicides |  |
| Expected Suicides |  |
| SMR |  |
| Male N |  |
| Male Observed Suicides |  |
| Male Expected Suicides |  |
| Male SMR |  |
| Female N |  |
| Female Observed Suicides |  |
| Female Expected Suicides |  |
| Female SMR |  |
| Cocaine |  |
| Subsample N |  |
| Observed Suicides |  |
| Expected Suicides |  |
| SMR |  |
| Male N |  |
| Male Observed Suicides |  |
| Male Expected Suicides |  |
| Male SMR |  |
| Female N |  |
| Female Observed Suicides |  |
| Female Expected Suicides |  |
| Female SMR |  |
| Amphetamines |  |
| Subsample N |  |
| Observed Suicides |  |
| Expected Suicides |  |
| SMR |  |
| Male N |  |
| Male Observed Suicides |  |
| Male Expected Suicides |  |
| Male SMR |  |
| Female N |  |
| Female Observed Suicides |  |
| Female Expected Suicides |  |
| Female SMR |  |
| Methamphetamines |  |
| Subsample N |  |
| Observed Suicides |  |
| Expected Suicides |  |
| SMR |  |
| Male N |  |
| Male Observed Suicides |  |
| Male Expected Suicides |  |
| Male SMR |  |
| Female N |  |
| Female Observed Suicides |  |
| Female Expected Suicides |  |
| Female SMR |  |
| Benzodiazepines |  |
| Subsample N |  |
| Observed Suicides |  |
| Expected Suicides |  |
| SMR |  |
| Male N |  |
| Male Observed Suicides |  |
| Male Expected Suicides |  |
| Male SMR |  |
| Female N |  |
| Female Observed Suicides |  |
| Female Expected Suicides |  |
| Female SMR |  |
| MDMA |  |
| Subsample N |  |
| Observed Suicides |  |
| Expected Suicides |  |
| SMR |  |
| Male N |  |
| Male Observed Suicides |  |
| Male Expected Suicides |  |
| Male SMR |  |
| Female N |  |
| Female Observed Suicides |  |
| Female Expected Suicides |  |
| Female SMR |  |
| Opioids: heroin, codeine, morphine |  |
| Subsample N |  |
| Observed Suicides |  |
| Expected Suicides |  |
| SMR |  |
| Male N |  |
| Male Observed Suicides |  |
| Male Expected Suicides |  |
| Male SMR |  |
| Female N |  |
| Female Observed Suicides |  |
| Female Expected Suicides |  |
| Female SMR |  |
| Synthetic Opioids |  |
| Subsample N |  |
| Observed Suicides |  |
| Expected Suicides |  |
| SMR |  |
| Male N |  |
| Male Observed Suicides |  |
| Male Expected Suicides |  |
| Male SMR |  |
| Female N |  |
| Female Observed Suicides |  |
| Female Expected Suicides |  |
| Female SMR |  |
| Mixed/Any Opioids |  |
| Subsample N |  |
| Observed Suicides |  |
| Expected Suicides |  |
| SMR |  |
| Male N |  |
| Male Observed Suicides |  |
| Male Expected Suicides |  |
| Male SMR |  |
| Female N |  |
| Female Observed Suicides |  |
| Female Expected Suicides |  |
| Female SMR |  |
| Inhalants |  |
| Subsample N |  |
| Observed Suicides |  |
| Expected Suicides |  |
| SMR |  |
| Male N |  |
| Male Observed Suicides |  |
| Male Expected Suicides |  |
| Male SMR |  |
| Female N |  |
| Female Observed Suicides |  |
| Female Expected Suicides |  |
| Female SMR |  |
| PCP |  |
| Subsample N |  |
| Observed Suicides |  |
| Expected Suicides |  |
| SMR |  |
| Male N |  |
| Male Observed Suicides |  |
| Male Expected Suicides |  |
| Male SMR |  |
| Female N |  |
| Female Observed Suicides |  |
| Female Expected Suicides |  |
| Female SMR |  |
| Mixed Substances |  |
| Subsample N |  |
| Observed Suicides |  |
| Expected Suicides |  |
| SMR |  |
| Male N |  |
| Male Observed Suicides |  |
| Male Expected Suicides |  |
| Male SMR |  |
| Female N |  |
| Female Observed Suicides |  |
| Female Expected Suicides |  |
| Female SMR |  |
| IV Drugs |  |
| Subsample N |  |
| Observed Suicides |  |
| Expected Suicides |  |
| SMR |  |
| Male N |  |
| Male Observed Suicides |  |
| Male Expected Suicides |  |
| Male SMR |  |
| Female N |  |
| Female Observed Suicides |  |
| Female Expected Suicides |  |
| Female SMR |  |
| Other: |  |
| Subsample N |  |
| Observed Suicides |  |
| Expected Suicides |  |
| SMR |  |
| Male N |  |
| Male Observed Suicides |  |
| Male Expected Suicides |  |
| Male SMR |  |
| Female N |  |
| Female Observed Suicides |  |
| Female Expected Suicides |  |
| Female SMR |  |
| Any Substance Use |  |
| Subsample N |  |
| Observed Suicides |  |
| Expected Suicides |  |
| SMR |  |
| Male N |  |
| Male Observed Suicides |  |
| Male Expected Suicides |  |
| Male SMR |  |
| Female N |  |
| Female Observed Suicides |  |
| Female Expected Suicides |  |
| Female SMR |  |
| Non-exposed Control Group |  |
| Subsample N |  |
| Observed Suicides |  |
| Expected Suicides |  |
| SMR |  |
| Male N |  |
| Male Observed Suicides |  |
| Male Expected Suicides |  |
| Male SMR |  |
| Female N |  |
| Female Observed Suicides |  |
| Female Expected Suicides |  |
| Female SMR |  |
| Substance Use Treatment |  |
| Alcohol |  |
| Subsample N |  |
| Observed Suicides |  |
| Expected Suicides |  |
| SMR |  |
| Male N |  |
| Male Observed Suicides |  |
| Male Expected Suicides |  |
| Male SMR |  |
| Female N |  |
| Female Observed Suicides |  |
| Female Expected Suicides |  |
| Female SMR |  |
| Tobacco |  |
| Subsample N |  |
| Observed Suicides |  |
| Expected Suicides |  |
| SMR |  |
| Male N |  |
| Male Observed Suicides |  |
| Male Expected Suicides |  |
| Male SMR |  |
| Female N |  |
| Female Observed Suicides |  |
| Female Expected Suicides |  |
| Female SMR |  |
| Cannabis |  |
| Subsample N |  |
| Observed Suicides |  |
| Expected Suicides |  |
| SMR |  |
| Male N |  |
| Male Observed Suicides |  |
| Male Expected Suicides |  |
| Male SMR |  |
| Female N |  |
| Female Observed Suicides |  |
| Female Expected Suicides |  |
| Female SMR |  |
| Cocaine |  |
| Subsample N |  |
| Observed Suicides |  |
| Expected Suicides |  |
| SMR |  |
| Male N |  |
| Male Observed Suicides |  |
| Male Expected Suicides |  |
| Male SMR |  |
| Female N |  |
| Female Observed Suicides |  |
| Female Expected Suicides |  |
| Female SMR |  |
| Amphetamines |  |
| Subsample N |  |
| Observed Suicides |  |
| Expected Suicides |  |
| SMR |  |
| Male N |  |
| Male Observed Suicides |  |
| Male Expected Suicides |  |
| Male SMR |  |
| Female N |  |
| Female Observed Suicides |  |
| Female Expected Suicides |  |
| Female SMR |  |
| Methamphetamines |  |
| Subsample N |  |
| Observed Suicides |  |
| Expected Suicides |  |
| SMR |  |
| Male N |  |
| Male Observed Suicides |  |
| Male Expected Suicides |  |
| Male SMR |  |
| Female N |  |
| Female Observed Suicides |  |
| Female Expected Suicides |  |
| Female SMR |  |
| Benzodiazepines |  |
| Subsample N |  |
| Observed Suicides |  |
| Expected Suicides |  |
| SMR |  |
| Male N |  |
| Male Observed Suicides |  |
| Male Expected Suicides |  |
| Male SMR |  |
| Female N |  |
| Female Observed Suicides |  |
| Female Expected Suicides |  |
| Female SMR |  |
| MDMA |  |
| Subsample N |  |
| Observed Suicides |  |
| Expected Suicides |  |
| SMR |  |
| Male N |  |
| Male Observed Suicides |  |
| Male Expected Suicides |  |
| Male SMR |  |
| Female N |  |
| Female Observed Suicides |  |
| Female Expected Suicides |  |
| Female SMR |  |
| Opioids: heroin, codeine, morphine |  |
| Subsample N |  |
| Observed Suicides |  |
| Expected Suicides |  |
| SMR |  |
| Male N |  |
| Male Observed Suicides |  |
| Male Expected Suicides |  |
| Male SMR |  |
| Female N |  |
| Female Observed Suicides |  |
| Female Expected Suicides |  |
| Female SMR |  |
| Synthetic Opioids |  |
| Subsample N |  |
| Observed Suicides |  |
| Expected Suicides |  |
| SMR |  |
| Male N |  |
| Male Observed Suicides |  |
| Male Expected Suicides |  |
| Male SMR |  |
| Female N |  |
| Female Observed Suicides |  |
| Female Expected Suicides |  |
| Female SMR |  |
| Mixed/Any Opioids |  |
| Subsample N |  |
| Observed Suicides |  |
| Expected Suicides |  |
| SMR |  |
| Male N |  |
| Male Observed Suicides |  |
| Male Expected Suicides |  |
| Male SMR |  |
| Female N |  |
| Female Observed Suicides |  |
| Female Expected Suicides |  |
| Female SMR |  |
| Inhalants |  |
| Subsample N |  |
| Observed Suicides |  |
| Expected Suicides |  |
| SMR |  |
| Male N |  |
| Male Observed Suicides |  |
| Male Expected Suicides |  |
| Male SMR |  |
| Female N |  |
| Female Observed Suicides |  |
| Female Expected Suicides |  |
| Female SMR |  |
| PCP |  |
| Subsample N |  |
| Observed Suicides |  |
| Expected Suicides |  |
| SMR |  |
| Male N |  |
| Male Observed Suicides |  |
| Male Expected Suicides |  |
| Male SMR |  |
| Female N |  |
| Female Observed Suicides |  |
| Female Expected Suicides |  |
| Female SMR |  |
| Mixed Substances |  |
| Subsample N |  |
| Observed Suicides |  |
| Expected Suicides |  |
| SMR |  |
| Male N |  |
| Male Observed Suicides |  |
| Male Expected Suicides |  |
| Male SMR |  |
| Female N |  |
| Female Observed Suicides |  |
| Female Expected Suicides |  |
| Female SMR |  |
| IV Drugs |  |
| Subsample N |  |
| Observed Suicides |  |
| Expected Suicides |  |
| SMR |  |
| Male N |  |
| Male Observed Suicides |  |
| Male Expected Suicides |  |
| Male SMR |  |
| Female N |  |
| Female Observed Suicides |  |
| Female Expected Suicides |  |
| Female SMR |  |
| Other: |  |
| Subsample N |  |
| Observed Suicides |  |
| Expected Suicides |  |
| SMR |  |
| Male N |  |
| Male Observed Suicides |  |
| Male Expected Suicides |  |
| Male SMR |  |
| Female N |  |
| Female Observed Suicides |  |
| Female Expected Suicides |  |
| Female SMR |  |
| Any Substance Use |  |
| Subsample N |  |
| Observed Suicides |  |
| Expected Suicides |  |
| SMR |  |
| Male N |  |
| Male Observed Suicides |  |
| Male Expected Suicides |  |
| Male SMR |  |
| Female N |  |
| Female Observed Suicides |  |
| Female Expected Suicides |  |
| Female SMR |  |
| Non-exposed Control Group |  |
| Subsample N |  |
| Observed Suicides |  |
| Expected Suicides |  |
| SMR |  |
| Male N |  |
| Male Observed Suicides |  |
| Male Expected Suicides |  |
| Male SMR |  |
| Female N |  |
| Female Observed Suicides |  |
| Female Expected Suicides |  |
| Female SMR |  |
| Do we need to extract expected suicide data from the WHO site? | Yes |
|  | No |
|  | Other: |

**Supplemental Table 3. Newcastle-Ottawa Scale**

| **Constructs** |  |
| --- | --- |
| Representativeness of the exposed cohort | Truly representative of the average person who uses substances in the community |
|  | Somewhat representative of the average person who uses substances in the community |
|  | Selected groups of users (e.g., nurses, volunteers, etc) |
|  | No description of the derivation of the cohort. |
|  | Supporting text: |
| Selection of the non-exposed cohort | Drawn from the same community as the exposed cohort |
|  | Drawn from a different source |
|  | No description of the non-exposed cohort |
|  | Supporting text: |
| Ascertainment of exposure | Secure record (e.g., surgical records) |
|  | Structured interview |
|  | Written self-report |
|  | No description |
|  | Supporting text: |
| Demonstration that outcome of interest (i.e., suicide) was not present at the start of the study | Yes |
|  | No |
|  | Supporting text: |
| Comparability of cohorts on the basis of the design or analysis | Study controls for history of suicide attempts |
|  | Study controls for mental health diagnoses |
|  | Study controls for both a history of suicide attempts and mental health diagnoses |
|  | Study does not control for any important factors |
|  | Supporting text: |
| Assessment of outcome | Independent blind assessment |
|  | Record linkage |
|  | Self-report |
|  | No description |
|  | Supporting text: |
| Was follow up long enough for outcomes to occur?  Study should be excluded if follow-up was less than 2 years. | Yes |
|  | No |
|  | Supporting text: |
| Adequacy of follow up cohorts  Study should be excluded if > 10% lost to follow up. | Complete follow up: all subjects accounted for |
|  | Subjects lost to follow up unlikely to introduce bias: small number lost or description provided of those who were lost |
|  | No description of those lost |
|  | No statement |
|  | Supporting text: |

**Supplemental Figure 1.** PRISMA Diagram^25^

**Identification**

Studies screened **(n = 10866)**

Studies sought for retrieval **(n = 205)**

Studies assessed for eligibility **(n = 205)**

References removed **(n = 6781)**

Duplicates identified manually (n = 6)

Duplicates identified by Covidence (n = 6775)

Marked as ineligible by automation tools (n = 0)

Other reasons (n = )

Studies excluded **(n = 10661)**

Studies not retrieved **(n = 0)**

Studies excluded **(n = 153)**

Not in English (n = 3)

Not suicide death (n = 17)

Not a cohort study (n = 2)

< 2 years of follow up (n = 10)

Not a longitudinal study (n = 1)

No general pop control group (n = 19)

Not focused on substance misuse (n = 35)

Required data not available for extraction (n = 67)

Duplicate not previously identified (n=4)

**Included**

Studies included in review **(n = 47)**

Included studies ongoing **(n = 0)**

Studies awaiting classification **(n = 0)**

**Screening**

Studies from databases/registers **(n = 17647)**

Embase (n = 1853)

CINAHL (n = 431)

PsycINFO (n = 375)

PubMed (n = 135)

MEDLINE (n = 126)

Unspecified (n = 14724)

**Supplemental Figure 2.** Individual and pooled SMR estimates for any substance use*


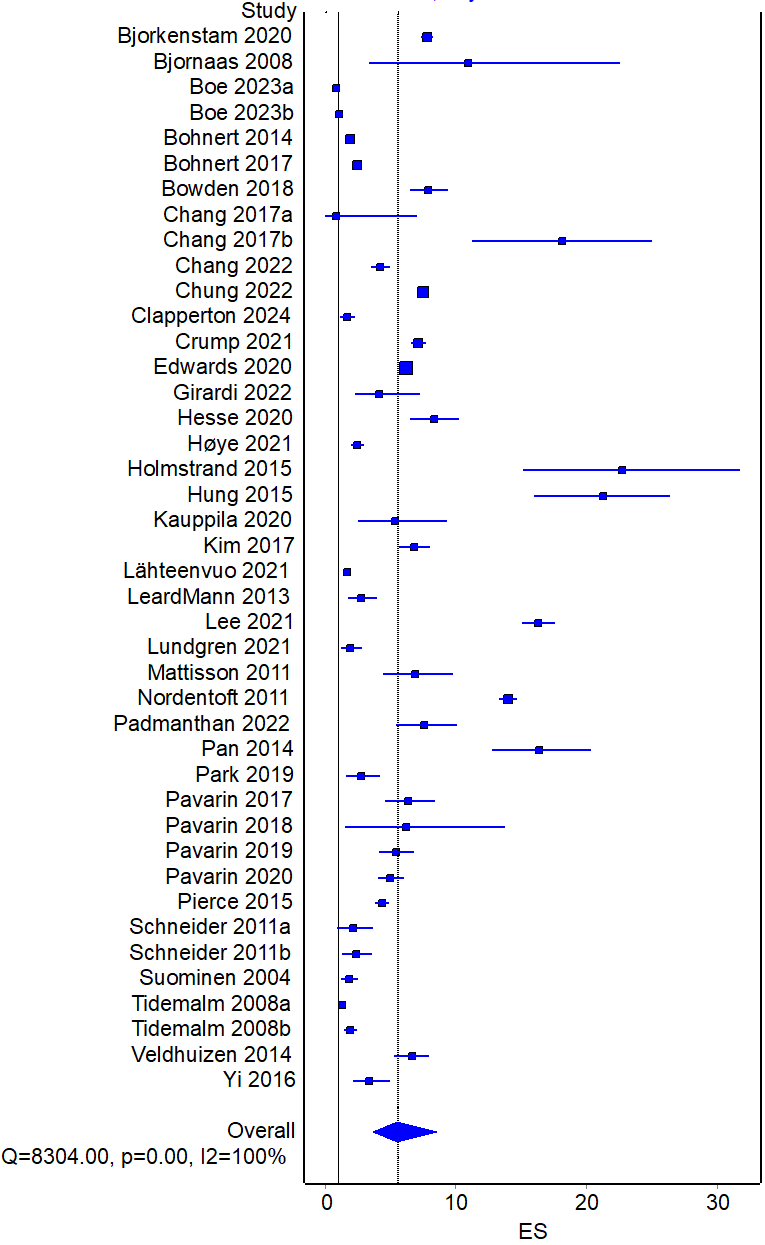


*One outlier excluded for ease of visualization: Pavarin 2008^58^ (ES 50, 0-196)

**Supplemental Figure 3**. Any/unspecified use of alcohol or tobacco


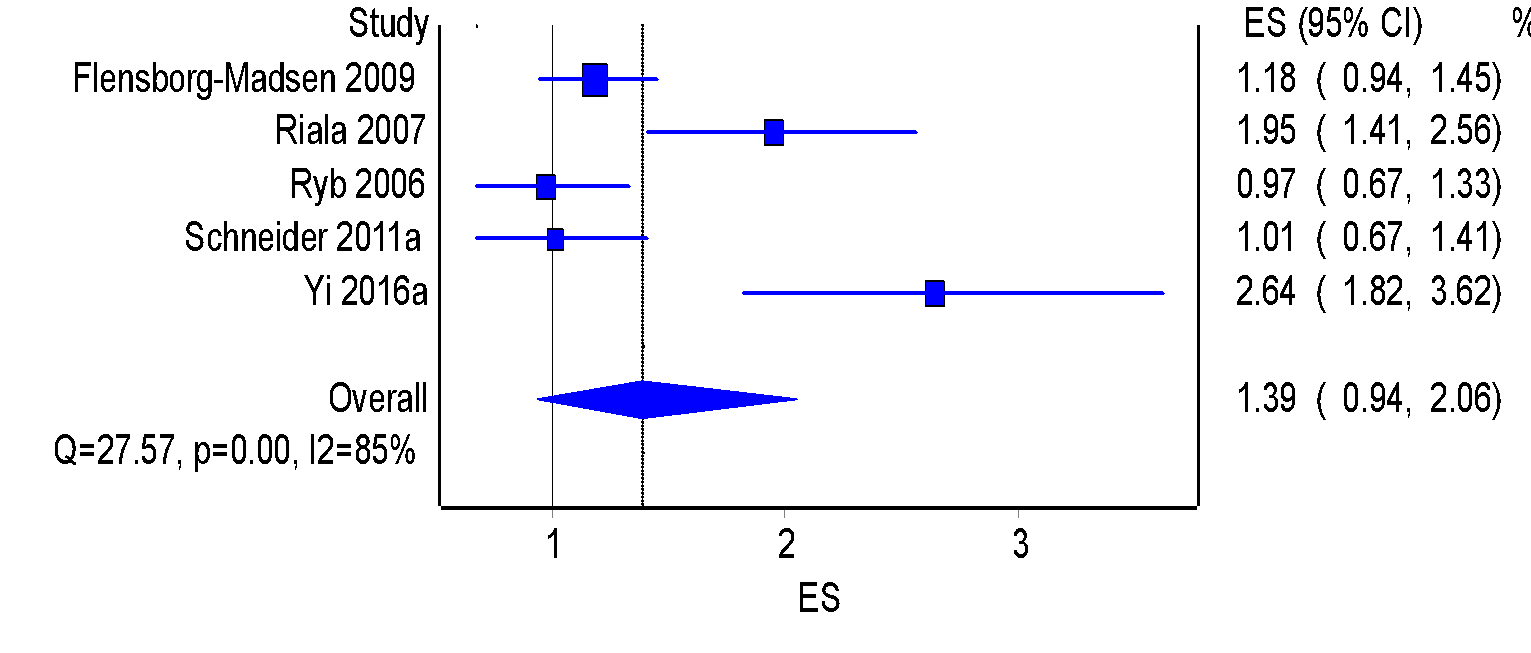


**Supplemental Figure 4**. Any substance use, stratified by sex


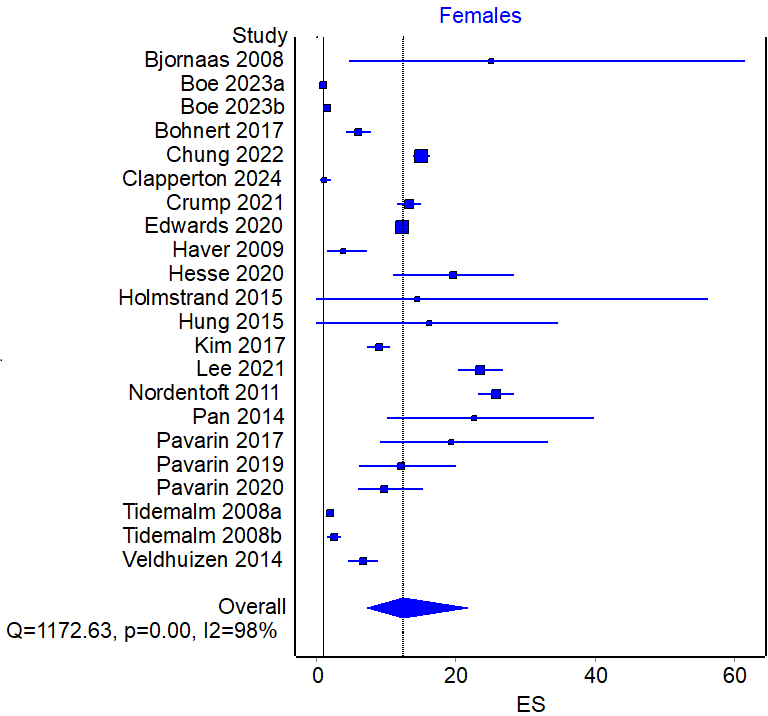


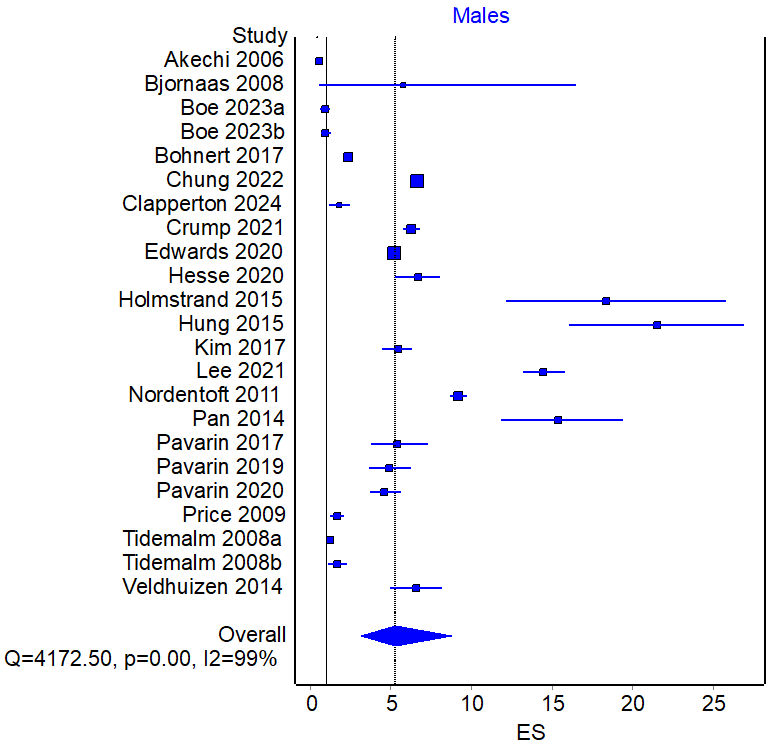


**Supplemental Figure 5.** Funnel plot of alcohol use overall and stratified by sex


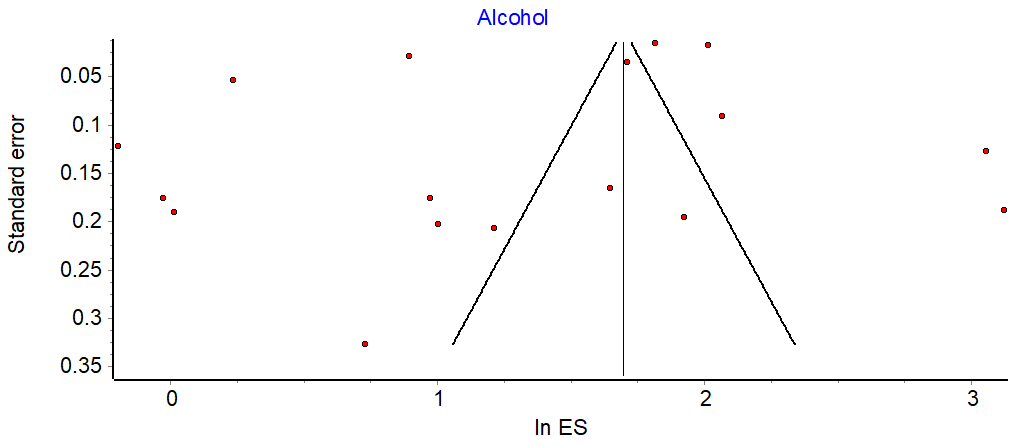


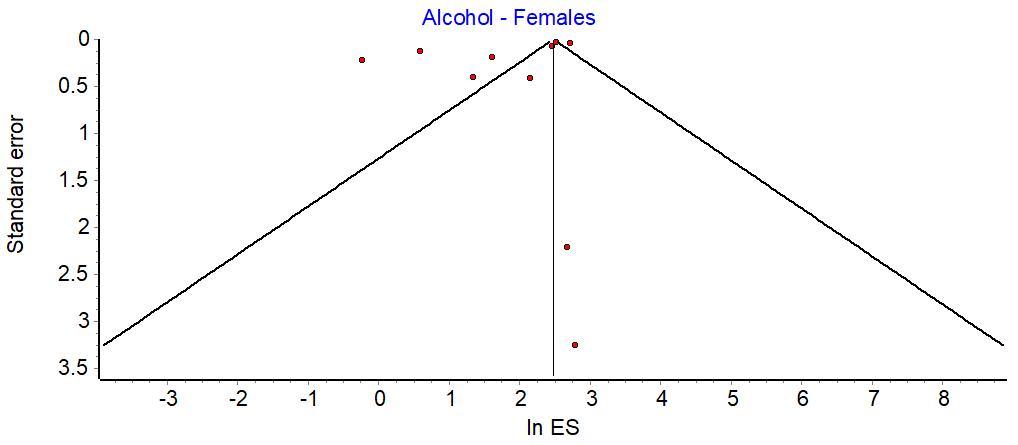


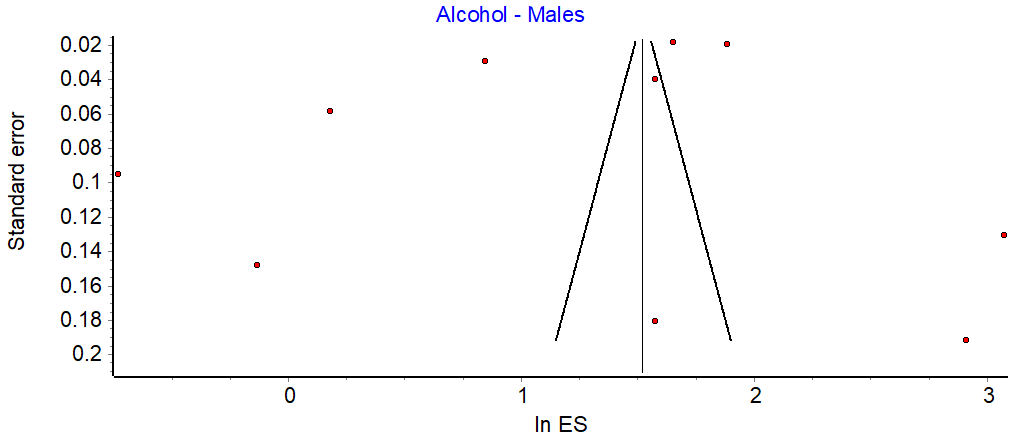


**Supplemental Figure 6.** Funnel plot of alcohol use and alcohol misuse

**
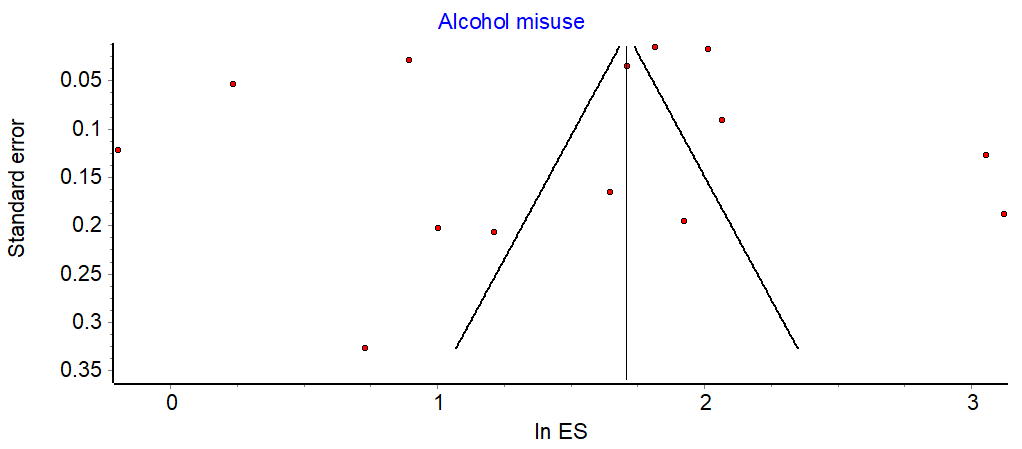
**

**
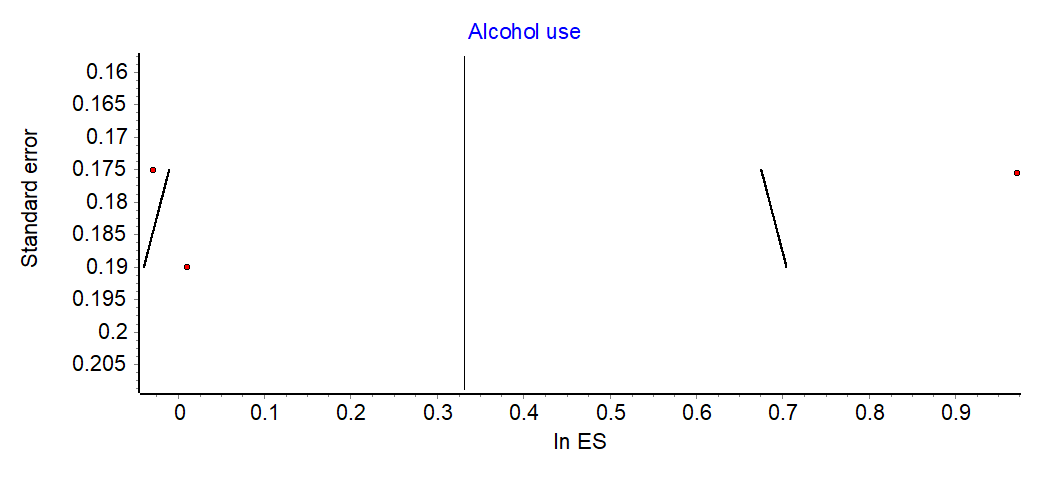
**

**Supplemental Figure 7.** Funnel plot of any substance use overall and stratified by sex


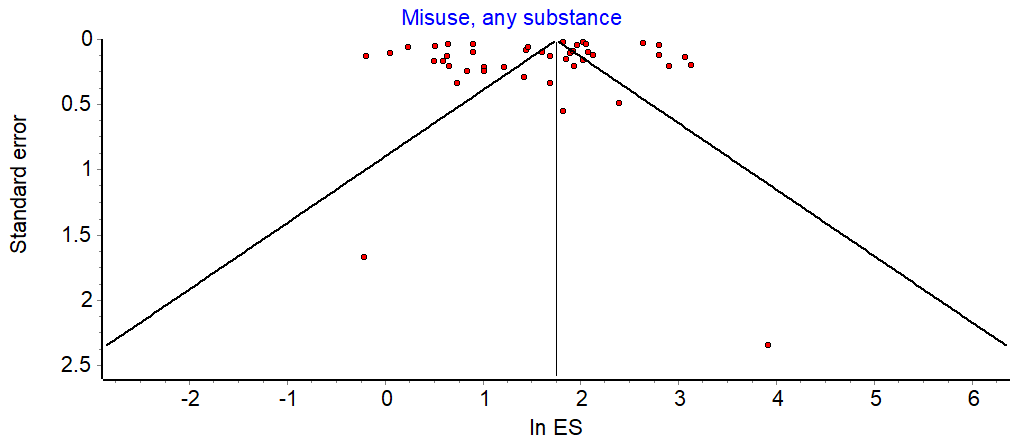


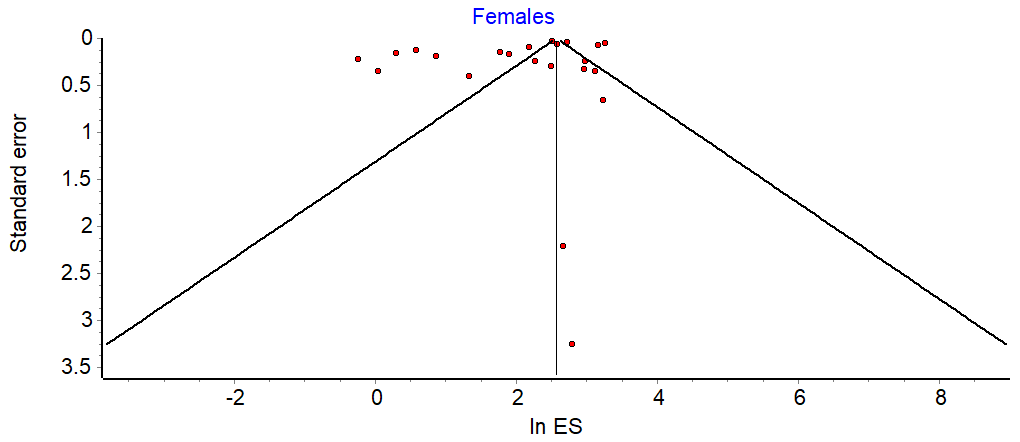


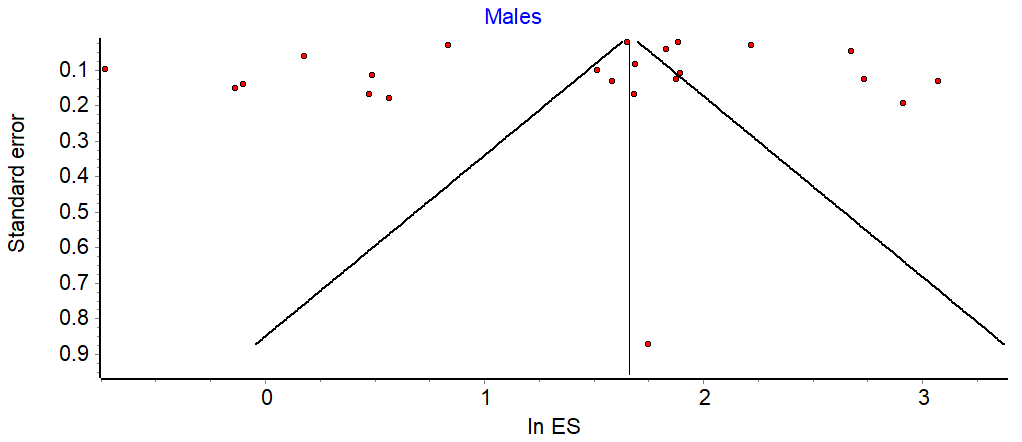


**Supplemental Figure 8.** Funnel plot of opioid use

**
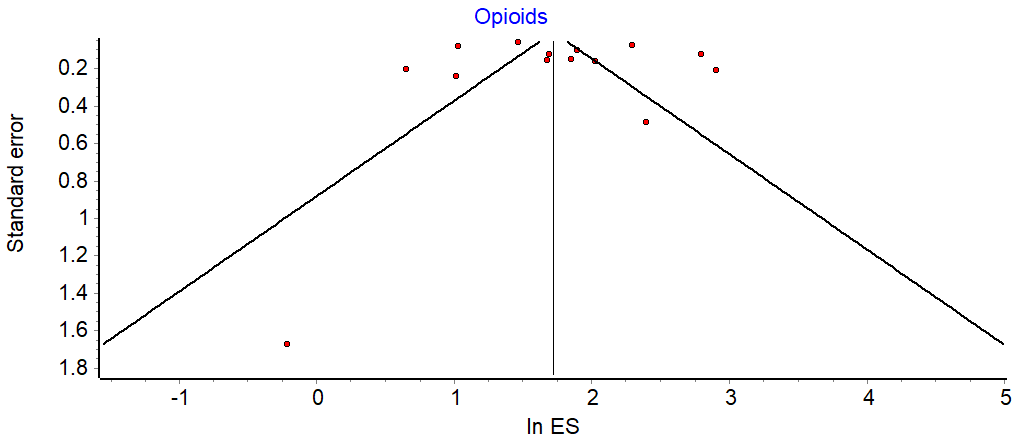
**

**Supplemental Figure 9.** Funnel plot of cannabis use

**
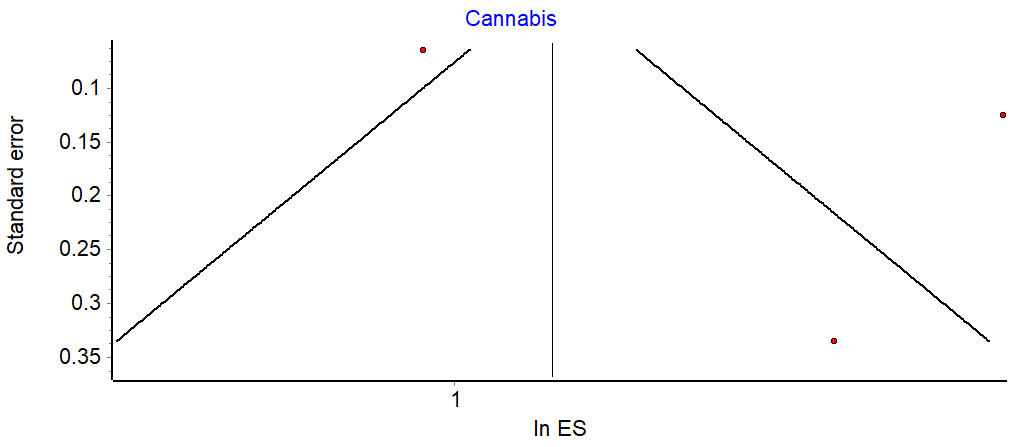
**

**Supplemental Figure 10.** Funnel plot of cocaine use


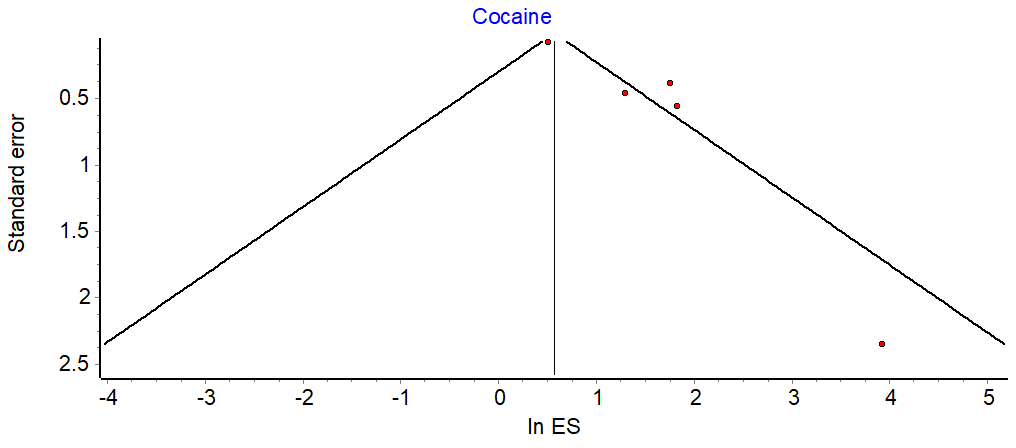


**Supplemental Figure 11.** Funnel plot of amphetamine use


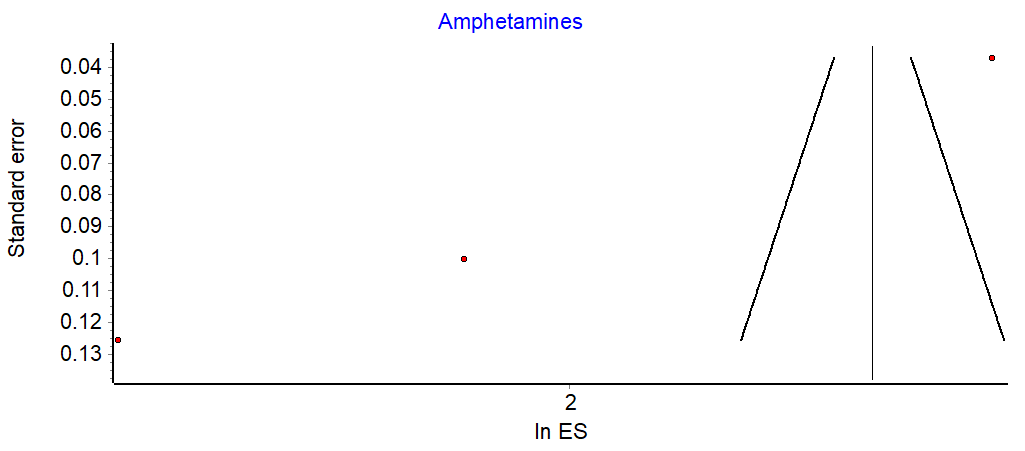


**Supplemental Figure 12.** Funnel plot of other types of substance use not previously included

**
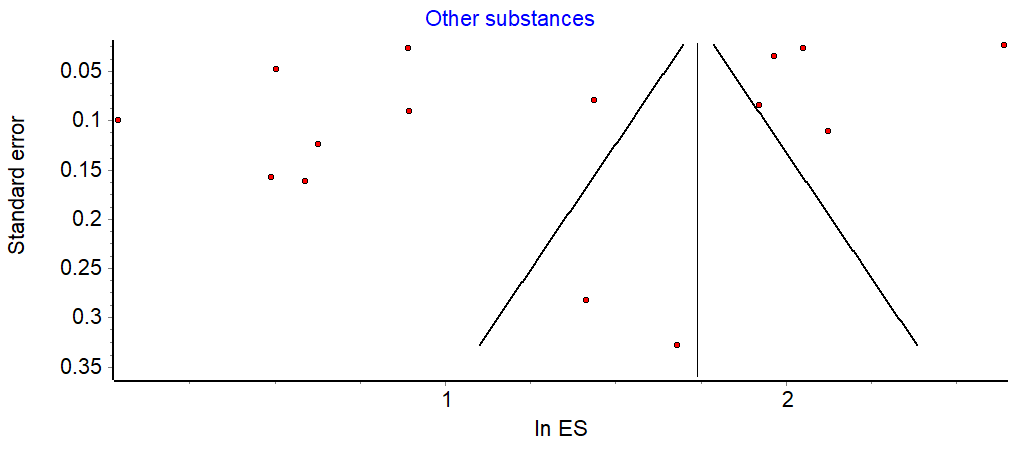
**
